# Pulsed electric fields-assisted extraction of valuable compounds from red grape pomace: Process optimization using response surface methodology

**DOI:** 10.3389/fnut.2023.1158019

**Published:** 2023-03-17

**Authors:** Serena Carpentieri, Giovanna Ferrari, Gianpiero Pataro

**Affiliations:** ^1^Department of Industrial Engineering, University of Salerno, Fisciano, SA, Italy; ^2^ProdAl Scarl - University of Salerno, Fisciano, SA, Italy

**Keywords:** pulsed electric fields (PEF), green extraction, red grape by-products, bioactive compounds, response surface methodology, HPLC-PDA

## Abstract

**Background:**

The application of Pulsed electric fields as a mild and easily scalable electrotechnology represents an effective approach to selectively intensify the extractability of bioactive compounds from grape pomace, one of the most abundant residues generated during the winemaking process.

**Objective:**

This study addressed the optimization of the pulsed electric fields (PEF)-assisted extraction to enhance the extraction yields of bioactive compounds from red grape pomace using response surface methodology (RSM).

**Methods:**

The cell disintegration index (Z_*p*_) was identified as response variable to determine the optimal PEF processing conditions in terms of field strength (E = 0.5–5 kV/cm) and energy input (W_T_ = 1–20 kJ/kg). For the solid-liquid extraction (SLE) process the effects of temperature (20–50°C), time (30–300min), and solvent concentration (0–50% ethanol in water) on total phenolic content (TPC), flavonoid content (FC), total anthocyanin content (TAC), tannin content (TC), and antioxidant activity (FRAP) of the extracts from untreated and PEF-treated plant tissues were assessed. The phenolic composition of the obtained extracts was determined *via* HPLC-PDA.

**Results:**

Results demonstrated that the application of PEF at the optimal processing conditions (E = 4.6 kV/cm, W_T_ = 20 kJ/kg) significantly enhanced the permeabilization degree of cell membrane of grape pomace tissues, thus intensifying the subsequent extractability of TPC (15%), FC (60%), TAC (23%), TC (42%), and FRAP values (31%) concerning the control extraction. HPLC-PDA analyses showed that, regardless of the application of PEF, the most abundant phenolic compounds were epicatechin, p-coumaric acid, and peonidin 3-O-glucoside, and no degradation of the specific compounds occurred upon PEF application.

**Conclusion:**

The optimization of the PEF-assisted extraction process allowed to significantly enhance the extraction yields of high-value-added compounds from red grape pomace, supporting further investigations of this process at a larger scale.

## 1. Introduction

Grape (*Vitis Vinifera*) is considered one of the major fruit crops in the world (1), with 75% of the global grape production used for winemaking (2–4).

World wine production in 2021, excluding juices and musts, is estimated at 260 MhL, with 19.3% produced by Italy, which represents the major wine producer. During the winemaking process, however, besides wine production, about 20–30% of the processed grapes is disposed of as wastes and by-products (5). Among these, grape pomace, a mixture of skins, pulp, seeds, and stalks, represents the most abundant by-product derived from the winemaking process (6). Moreover, along with the continuous expansion of the global wine sector, which is expected to almost double by 2028 (7), the concurrent increase in grape pomace production is predictable.

This further enhances the concern regarding the disposal of these matrices, thus pushing the wineries to move toward more sustainable production practices and valorisation of grape by-products.

Nevertheless, grape pomace has been underexploited so far, being usually used for low-value-added applications (8). In particular, it has been estimated that currently about 3% of grape pomace is reused for animal feed, or as waste-based compost, while the majority is disposed of in landfills, increasing the negative impact on the environment (9).

However, grape pomace still retains appreciable amounts of valuable compounds, constituting a promising source of bioactive compounds such as anthocyanins, flavonoids, and tannins with nutritional properties and health-beneficial effects (3, 10, 11), due to their anti-inflammatory potential (12), antioxidant and antimicrobial capacities (8, 13), and prebiotic activity (14). These invaluable properties are increasingly supporting the idea of valorising low-cost grape processing by-products, namely grape pomace, through the sustainable recovery of natural ingredients and nutraceuticals (2).

To effectively recover these valuable intracellular compounds from plant tissues, an efficient solid-liquid extraction (SLE) process is necessary. Generally, the process conducted by conventional SLE techniques is hampered by the presence of a physical barrier (cell membranes and wall) limiting the diffusion of solvent and intracellular compounds through the cell envelope (15–17).

Due to the intrinsic limitations associated with conventional SLE techniques, many researchers are striving to investigate the usage of cell disruption pre-treatment techniques that can help in intensifying the recovery of target compounds from plant biomasses, by reducing the mass transfer resistance through the cell envelope (2, 17). In this line, the application of pulsed electric fields (PEF) treatment prior to SLE is gaining great interest as a mild, energy-efficient and scalable cell disruption technology (18).

Briefly, during the PEF treatment the wet plant matrix, placed between two electrodes, is exposed to a train of short duration pulses (1 μs – 1 ms) of relatively low electric field strength (0.5–10 kV/cm) and total specific energy input (1–20 kJ/kg), resulting in the permeabilization of cell membranes (2). The application of this electrotechnology has been demonstrated to be an effective approach to selectively recover intracellular valuable compounds from various agri-food by-products, including those generated during the winemaking process, and especially grape pomace and skins (19–23), with reduced energy costs, solvent consumption, and treatment time.

However, as already demonstrated in a recent study (2) focused on the optimization of PEF-assisted extraction of phenolic compounds from white grape pomace, an optimization step of both PEF pre-treatment and the subsequent SLE process is necessary to fully exploit the possible benefits of PEF-assisted extraction process compared to the conventional SLE. To date, only a few studies (24–27) addressed the optimization of the variables (diffusion time, solid-liquid ratio, temperature, type and concentration of solvent) involved in the conventional extraction process of bioactives from red grape by-products through response surface methodology (RSM), a statistical tool that includes optimization procedures for the settings of input variables that affect targeted response variables (28). Moreover, as per literature survey, very little attention has been paid on the optimization of the whole extraction process of valuable compounds from grape residues assisted by emerging technologies (2, 29, 30), but none of them was addressed to the optimization of the PEF-assisted extraction process of bioactive compounds from red grape pomace. In particular, Carpentieri et al. (2), using the same equipment and extraction protocols used in the present work, studied the optimization of PEF-assisted extraction of phenolic compounds from white grape pomace. The latter, however, are typically generated after the crushing and pressing steps of grape, without the intermediate maceration/fermentation step required for the production of red wine, which could further affect the cell integrity and, consequently, the behavior of pomace toward the subsequent PEF treatment.

The main aim of the present research was to investigate the potential of PEF technology to enhance the extraction yield of intracellular valuable compounds, such as total phenolic compounds, flavonoids, tannins, and anthocyanins from red grape pomace, and to optimize the PEF-assisted extraction process. PEF processing conditions, expressed in terms of different combinations of field strength (E) and energy input (W_T_), were optimized by RSM in order to determine the less severe treatment conditions that led to the highest cell disintegration index (*Z*_*p*_) of grape pomace tissues. RSM was also used to optimize the solid-liquid extraction (SLE) process of the target bioactive compounds from grape pomace by investigating the effect of ethanol concentration, diffusion time, and extraction temperature, during either conventional or PEF-assisted extraction process. Then, the effect of PEF-assisted extraction on the composition of the phenolic compounds in the obtained extracts was determined by HPLC-PDA analyses.

## 2. Materials and methods

### 2.1. Chemicals and raw materials

Ethanol and all chemicals and standards involved in the analyses were supplied by Sigma Aldrich (Steinheim, Germany).

Red grape pomace, mainly composed of skins and seeds, was provided by a local winery (Sabino Urciuoli S.a.s, Avellino, Italy). The pomace were generated during the vinification process of “Aglianico” grape variety in 2021, and collected at the end of crushing, 10 days of maceration and pressing steps. The collected samples were stored under refrigerated conditions (T = 4°C) until use.

### 2.2. PEF equipment

Fresh grape pomace underwent PEF pre-treatments in a laboratory-scale batch system, previously described elsewhere (31). Briefly, the system was equipped with an electric pulse generator (25 kV-500 A, Modulator PG, ScandiNova, Uppsala, Sweden), able to deliver monopolar square wave pulses with different pulse width (3–25 μs) and frequency (1–450 Hz) to the plant tissues placed between the electrodes of a parallel plate treatment chamber (electrode area 7.1 cm^2^, electrode gap up to 5 cm). High voltage and current probes, connected to an oscilloscope, were used to measure the actual voltage and current passing through the chamber. The maximum electric field intensity (E, in kV/cm) and the total specific energy input (W_T_, in kJ/kg of plant tissues) were calculated as reported by Carpentieri et al. (2).

### 2.3. Cell membrane electroporation induced by PEF

In order to quantify the permeabilization degree of the cell membrane of grape pomace tissue upon PEF treatment, the cell disintegration index (*Z*_*p*_) was evaluated *via* impedance analyses by measuring the electrical complex impedance in frequency sweep (10^2^-10^6^ Hz) of untreated and PEF-treated samples, as described in detail by Carpentieri et al. (2). For each measurement, approximately 5 g of sample were loaded into the measuring cell and exposed to a given combination of field strength (E = 0.5–5 kV/cm) and energy input (W_T_ = 1 - 20 kJ/kg) at a constant pulse width (20 μs) and frequency (5 Hz). For each treatment condition investigated, the *Z*_*p*_ value, ranging from 0 (for intact tissue) to 1 (for fully permeabilized tissue), was calculated using Equation (1) (32):


(1) $$\begin{array}{l} Z_{p}=\;\frac{\left|Z_{untr\;\left(0,1\;kHz\right)}|-|Z_{tr\;\left(0,1\;kHz\right)}\right|}{\left|Z_{untr\;\left(0,1\;kHz\right)}|-|Z_{tr\;\left(1\;MHz\right)}\right|} \end{array}$$

where |*Z*_*untr*_| and |*Z*_*tr*_| denote, respectively, the absolute values of the complex impedance of untreated and PEF treated tissue detected in the low (0.1 kHz) and high (1 MHz) frequency ranges.

All the measurements were carried out in triplicate.

The obtained *Z*_*p*_ values were used to determine the optimal treatment conditions in terms of electric field strength (*E*_*opt*_) and total specific energy input (*W*_*T,opt*_), that maximize the cell membrane permeabilization degree with the minimum treatment severity (2, 32, 33).

### 2.4. PEF-assisted extraction

For each processing condition investigated, approximately 5 g of grape pomace were loaded into the treatment chamber and PEF-treated at the previously determined optimal conditions (*E*_*opt*_*, W*_*T,opt*_). After the PEF treatment, the samples were immediately transferred into 100 mL Pyrex flasks where an ethanol-water mixture was added at a solid to liquid ratio of 1:10 g/mL. The flasks were then introduced in an orbital incubator S150 (PBI International Milan, Italy) and the extraction started. The process was carried out under constant shaking at 160 rpm for different times (0–300 min), temperatures (20–50°C), and ethanol concentration (0–50%). The same experimental design and extraction conditions were used for untreated (control) samples undergoing conventional SLE without the application of PEF pre-treatment.

The extracts, after centrifugation at 5289xg (PK130R model, ALC International, Cologno Monzese, IT) for 10 min at 4°C, were stored at 4°C until analyzed.

### 2.5. Experimental design

Response surface methodology was used to gain insight into the significance of the input factors on the response variables, as well as to determine optimal parameters of PEF pre-treatment maximizing the *Z*_*p*_ value, and optimal conditions of the SLE process leading to the highest extraction yields of total phenolic content (TPC), flavonoid content (FC), total anthocyanin content (TAC), tannin content (TC), and antioxidant activity of grape pomace extracts from untreated (control) and PEF-treated samples.

A three-factors face-cantered central composite design (FC-CCD) was used to determine how the electric field strength (X_1_, 0.5–5 kV/cm) and total specific energy input (X_2_, 1–20 kJ/kg) affected the level of cell membrane permeabilization of grape pomace tissues upon PEF pre-treatment. The obtained experimental design is made up of 9 runs (Table 1), with the Z_*p*_ (Y_1_) of PEF-treated samples considered as response variable. The obtained data were modeled with the second-order polynomial model reported in Equation (2):


(2) $$\begin{array}{l} Y_{k}=\beta_{0}+\overset{2}{\underset{i=1}{\sum }}\beta_{i}X_{i}+\overset{2}{\underset{i=1}{\sum }}\beta_{ii}X_{i}^{2}+\overset{2}{\underset{i=1}{\sum }}\overset{3}{\underset{j=i+1}{\sum }}\beta_{ij}X_{i}X_{j} \end{array}$$

where Y_k_ is the response variable; X_i_ and X_j_ are the independent factors; β_0_, β_i_, β_ii_, and β_ij_ are the intercept, and regression coefficients of the linear, quadratic, and interaction terms of the model, respectively.

**Table 1:** Effect of of the two independent factors (E, W_T_) investigated on the response variable (*Z*_*p*_) of the PEF treated grape pomace tissues according to the FC-CCD.

**Run**	**Independent variables**		**Response**
	**E (kV/cm)**	**W**_T_ **(kJ/kg)**	***Z**_*p*_*
1	0.5	1	0.033 ± 0.003a
2	0.5	10.5	0.075 ± 0.002b
3	0.5	20	0.130 ± 0.015c
4	2.75	1	0.208 ± 0.034d
5	2.75	10.5	0.513 ± 0.020f
6	2.75	20	0.580 ± 0.010g
7	5.0	1	0.417 ± 0.009e
8	5.0	10.5	0.521 ± 0.010f
9	5.0	20	0.700 ± 0.007h

The results are expressed as mean ± standard deviation (n = 2 for factorial and axial points, n = 5 for central point). Values with different lowercase letter are significantly different (p ≤ 0.05).

The same experimental design was used to analyze the effect of ethanol concentration (X_3_, 0–50%, v/v) in a water-ethanol solvent mixture, extraction time (X_4_, 30–300 min), and extraction temperature (X_5_, 20–50°C) on the response variables, namely total phenolic content (Y_2_), flavonoid content (Y_3_), antioxidant activity (Y_4_), anthocyanin content (Y_5_), tannin content (Y_6_), of untreated and PEF-treated samples. The experimental design consisted of 15 runs including five replicates of central points (Tables 2, 3). A two-factor interaction (2FI) model reported in Equation (3) was applied to predict the response variables as function of the investigated independent factors:


(3) $$\begin{array}{l} Y_{k}=\alpha_{0}+\overset{3}{\underset{i=1}{\sum }}\alpha_{i}X_{i}+\overset{3}{\underset{i=1}{\sum }}\overset{4}{\underset{j=i+1}{\sum }}\alpha_{ij}X_{i}X_{j}+\overset{3}{\underset{i=1}{\sum }}\overset{5}{\underset{j=i+2}{\sum }}\alpha_{ij}X_{i}X_{j} \end{array}$$

**Table 2:** Effect of the independent factors investigated on the response variables (TPC, FC, and FRAP) in grape pomace extracts from untreated and PEF (4.6 kV/cm, 20 kJ/kg)-treated samples.

**Run**	**Variables**			**SLE**			**PEF-assisted extraction**		
	**Ethanol (%)**	**t (min)**	**T (**°**C)**	**TPC**	**FC**	**FRAP**	**TPC**	**FC**	**FRAP**
1	0	30	20	0.02 ± 0.01a	2.44 ± 0.10a	0.69 ± 0.01a	0.30 ± 0.06b	3.94 ± 0.23b	0.75 ± 0.01b
2	0	300	20	0.12 ± 0.11a	4.37 ± 0.54a	0.68 ± 0.03a	0.19 ± 0.15a	13.26 ± 0.50b	0.70 ± 0.02a
3	25	165	20	1.11 ± 0.22a	7.03 ± 0.54a	0.44 ± 0.04a	1.17 ± 0.07a	7.92 ± 0.44a	0.47 ± 0.01a
4	50	30	20	1.63 ± 0.19a	7.25 ± 0.84a	0.67 ± 0.01a	1.78 ± 0.04b	7.35 ± 0.14a	0.70 ± 0.04a
5	50	300	20	2.42 ± 0.20a	13.03 ± 0.72a	0.99 ± 0.10a	2.65 ± 0.26a	14.11 ± 0.21b	1.21 ± 0.02b
6	0	165	35	0.56 ± 0.10a	7.11 ± 0.91a	1.17 ± 0.03a	0.77 ± 0.28a	9.37 ± 0.04b	1.92 ± 0.03b
7	25	30	35	0.70 ± 0.14a	5.27± 0.42a	0.20 ± 0.04a	0.94 ± 0.07b	7.25 ± 0.44b	0.34 ± 0.01b
8	25	165	35	1.93 ± 0.10a	10.26 ± 0.04a	1.05 ± 0.02a	2.77 ± 0.56b	17.13 ± 0.08b	1.08 ± 0.04a
9	25	300	35	2.49 ± 0.50a	16.01 ± 0.09a	0.88 ± 0.05a	2.91 ± 0.22a	23.78 ± 0.45b	1.11 ± 0.03a
10	50	165	35	5.20 ± 0.11a	19.98 ± 0.21a	2.28 ± 0.22a	5.39 ± 0.07a	26.22 ± 0.94b	2.72 ± 0.10b
11	0	30	50	0.31 ± 0.06a	3.43 ± 0.22a	0.84 ± 0.08a	0.44 ± 0.04b	7.08 ± 0.34b	0.92 ± 0.02a
12	0	300	50	1.25 ± 0.07a	13.40 ± 0.06a	1.22 ± 0.07a	1.39 ± 0.21a	15.05 ± 0.63b	1.23 ± 0.10a
13	25	165	50	3.81 ± 0.10a	28.40 ± 0.84a	2.09 ± 0.10a	4.37 ± 0.21b	29.40 ± 0.34a	2.10 ± 0.05a
14	50	30	50	3.76 ± 0.14a	12.92 ± 0.17a	1.48 ± 0.09a	6.30 ± 0.23b	18.48 ± 0.42b	2.03 ± 0.02b
15	50	300	50	8.30 ± 0.11a	36.68 ± 0.48a	4.58 ± 0.15a	9.51 ± 0.02b	58.53± 0.39b	5.99 ± 0.12b

TPC is expressed in mg GAE/g_DM_ grape pomace, FC is expressed in mg QE/g_DM_ grape pomace and FRAP in mg AAE/g_DM_ grape pomace. The results are expressed as mean ± standard deviation (n = 2 for factorial and axial points, n = 5 for central point). Ethanol, means ethanol percentage in ethanol-water mixture (%, v/v); t, extraction time (min) T, extraction temperature (°C). Values of the same dependent variable with different lowercase letter within the same row are significantly different (p ≤ 0.05).

**Table 3:** Effect of the independent factors investigated on the response variables (TAC, TC) in grape pomace extracts from untreated and PEF (4.6 kV/cm, 20 kJ/kg)-treated samples.

**Run**	**Variables**			**SLE**		**PEF-assisted extraction**	
	**Ethanol (%)**	**t (min)**	**T (**°**C)**	**TAC**	**TC**	**TAC**	**TC**
1	0	30	20	0.022 ± 0.00a	0.003 ± 0.00a	0.025 ± 0.00b	0.005 ± 0.00b
2	0	300	20	0.038 ± 0.01a	0.21 ± 0.01a	0.041 ± 0.05b	0.22 ± 0.01a
3	25	165	20	0.15 ± 0.02b	0.29 ± 0.02a	0.12 ± 0.01a	0.31 ± 0.01a
4	50	30	20	0.25 ± 0.02a	0.66 ± 0.01a	0.30 ± 0.01b	0.70 ± 0.03b
5	50	300	20	0.41 ± 0.01a	0.93 ± 0.01a	0.44 ± 0.01b	1.65 ± 0.10b
6	0	165	35	0.031 ± 0.00a	0.15 ± 0.02a	0.050 ± 0.00b	0.26 ± 0.03b
7	25	30	35	0.10 ± 0.0a	0.31 ± 0.10a	0.12 ± 0.10b	0.52 ± 0.12a
8	25	165	35	0.12 ± 0.01a	0.66 ± 0.01a	0.32 ± 0.02b	1.62 ± 0.20b
9	25	300	35	0.24 ± 0.02a	1.28 ± 0.10a	0.34 ± 0.03b	2.24 ± 0.20b
10	50	165	35	0.71 ± 0.05a	3.45 ± 0.30a	0.78 ± 0.03b	3.19 ± 0.01a
11	0	30	50	0.035 ± 0.00a	0.18 ± 0.01a	0.045 ± 0.00b	0.10 ± 0.01b
12	0	300	50	0.037 ± 0.01a	0.40 ± 0.02a	0.060 ± 0.01b	1.21 ± 0.11b
13	25	165	50	0.56 ± 0.10a	1.17 ± 0.15a	0.36 ± 0.10a	1.68 ± 0.20b
14	50	30	50	0.23 ± 0.01a	1.77 ± 0.10a	0.46 ± 0.05b	2.10 ± 0.30a
15	50	300	50	0.84 ± 0.05a	3.84 ± 0.05a	1.03 ± 0.06b	5.45 ± 0.15b

TAC is expressed in mg C3G/g_DM_ grape pomace, TC is expressed in mg TC/g_DM_ grape pomace. The results are expressed as mean ± standard deviation (n = 2 for factorial and axial points, n = 5 for central point). Ethanol, means ethanol percentage in ethanol-water mixture (%, v/v); t, extraction time (min) T, extraction temperature (°C). Values of the same dependent variable with different lowercase letter within the same row are significantly different (p ≤ 0.05).

where Y_k_ is the predicted response variables; X_i_ and X_j_ are the independent factors; α_0_, α_i_, and α_ij_ are the intercept, and regression coefficients of the linear, and interaction terms of the model, respectively.

### 2.6. Extracts characterization

#### 2.6.1. Total phenolic content

The total phenolic content (TPC) of the obtained extracts was determined using the Folin-Ciocalteau method as reported by Carpentieri et al. (2). Gallic acid dissolved in ethanol/water mixtures (0–50%, v/v) was used as the standard for the calibration curve in a concentration range of 1–100 mg/L. Results were expressed as milligrams of gallic acid equivalents (GAE) per g of dry weight (g_DM_) grape pomace.

#### 2.6.2. Flavonoid content

The flavonoid content (FC) of the obtained extracts was determine using the Aluminum-chloride colorimetric assay as previously described by Carpentieri et al. (2). Quercetin dissolved in ethanol/water mixtures (0–50%, v/v) was used as the standard for the calibration curves in a concentration range of 20–100 mg/L. The obtained results were expressed as mg of quercetin equivalent (QE) per g_DM_ of grape pomace.

#### 2.6.3. Evaluation of TAC

The total anthocyanin content (TAC) of the obtained extracts was determined using the pH differential method described by Lee et al. (34) with slight modifications.

Briefly, two mixtures were prepared per each extract from untreated and PEF-treated grape pomace by diluting, with a dilution factor equal to 5, one sample with pH 1.0 buffer (0.19% (w/v) of potassium chloride in water, and the other with pH 4.5 buffer (5.44% (w/v) of sodium acetate in water. The absorbance of the diluted reacting solutions was then measured at 520 and 700 nm using the V-650 spectrophotometer within 30 min from their preparation.

Results were determined by means of the following formula and expressed as mg of C3G (cyanidin-3-glucoside) per g_DM_ of grape pomace:


(4) $$\begin{array}{l} C=\frac{A^{*}MW^{*}DF^{*}10^{3}}{\varepsilon }\;*\;\frac{L}{S}\;*\;\frac{m_{TOT}}{m_{DW}} \end{array}$$

where:

*A* = (*A*_520n*m*_ – *A*_700n*m*_)_= 1_ – (*A*_520n*m*_ – *A*_700 n*m*_)_p*H* = 4.5_;

MW (molecular weights of cyanidin-3-glucoside) = 449.2 *g/mol*;

DF = dilution factor;

ε = (molar extinction coefficient) = 26900 *L*/*mol*/*cm*;

10^3^ = conversion factor from g to mg;

L/S = liquid-to-solid ratio;

m_TOT_/m_DW_ = ratio between the total mass of fresh grape pomace and the mass of dry grape pomace.

#### 2.6.4. Evaluation of tannin content

The total tannin content was determined according to the colorimetric method described by Tempel (35) with slight modifications. Briefly, 2 mL of distilled water and 6 mL of concentrated HCl were added to 4 mL of the extract. The same solution was prepared twice in two separate vials. Afterwards, one of the two vials was heated up to 100°C for 30 min and then cooled down. Then an amount equal to 1 mL of EtOH at 95% was added and the absorbance of the obtained sample was read at 550 nm. The concentration of tannins, expressed as mg of tannin content per g_DM_ of grape pomace was calculated by using the following formula:


(5) $$\begin{array}{l} C=19.33*\text{Δ}D*\frac{L}{S}*\frac{m_{TOT}}{m_{DW}} \end{array}$$

where Δ*D* = *D*2 – *D*1, with *D1* being the absorbance of the unheated vial, and *D2* the absorbance of the heated vial.

#### 2.6.5. Ferric reducing antioxidant power

FRAP assay of grape pomace extracts was performed according to the method reported by Benzie and Strain (36) with slight modifications, as thoroughly described elsewhere (2). Ascorbic acid dissolved in ethanol/water mixtures (0–50%, v/v) was used as the standard for the calibration curves in a concentration range comprised between 0 and 2 mmol/L. The antioxidant capacity was expressed as mg of ascorbic acid equivalents (mg AAE) per g_DM_ of grape pomace.

#### 2.6.6. HPLC-PDA analyses of the extracts

The identification of the most abundant bioactive compounds of the extracts from untreated and PEF-treated grape pomace was performed by High-Performance Liquid Chromatography - Photodiode Array Detection (HPLC-PDA) analyses, according to the method described in detail by Carpentieri et al. (2). A Waters 1525 Separation Module equipped with a photodiode array detector Water 2996 (Waters Corporation, USA) was used. Analytical separation was carried out using a Waters Spherisorb C18 reverse phase column (5 μm ODS2, 4,6 mm × 250 mm, Water Corporation, USA). The quantification of each phenolic compound was carried out at the wavelength of its maximum absorbance (λ), namely 271 nm for gallic acid, 320 nm for chlorogenic acid, caffeic acid and p-coumaric acid, 280 nm for epicatechin and phlorizin, 283 nm for naringin, and 260 nm for rutin. The commercial standards were dissolved into the extraction solvent to generate standard calibration curves (R^2^ = 0.998). The results were expressed as mg of the target compound/g_DM_ of grape pomace.

The same equipment was utilized for the identification of anthocyanins in the grape pomace extracts by following the method described by Lee et al. (37). The mobile phase consisted of (A) 100%, v/v acetonitrile, and (B) 10%, v/v acetic acid and 1%, v/v phosphoric acid in water. The injection volume and the flow rate of the mobile phase were 25 μL and 1.0 mL/min, respectively. A linear gradient consisting of 0–25 min from 2 A to 20% A, and 25–30 min from 20 A to 40% A was used, with simultaneous detection at the wavelength of 280 and 520 nm. The commercial standard was dissolved into the extraction solvent [ethanol/water mixtures (0–50%, v/v)] to generate a standard calibration curve (R^2^ = 0.988). The results were expressed as mg of the target anthocyanin/g_DM_ of grape pomace.

### 2.7. Statistical analysis

All the experiments and analyses were performed in triplicate and the results are means ± SD. One-way variance (ANOVA) and Tukey test performed by using SPSS 20 (SPSS IBM., Chicago, USA) statistical package was used to determine the significant (p < 0.05) of differences the among mean values. The FC-CCD design and the analysis of the data were performed using the software package Design Expert Version 12 software (Minneapolis, MN). Five replicates of the optimal conditions were performed to validate the models.

## 3. Results and discussion

### 3.1. Permeabilization degree of cell membrane of grape pomace tissue upon PEF treatment

The effect of the PEF treatment on the degree of cell membrane permeabilization of red grape pomace tissue was investigated *via* complex electrical impedance measurements of untreated and PEF-treated samples. The data of the absolute values of the complex electrical impedance of untreated and PEF-treated samples as a function of the electrical frequency and at different field strength E (0.5–5 kV/cm) and specific energy input W_T_ (1-20 kJ/kg) are plotted in Supplementary Figure S1. This data was used for the evaluation of the cell disintegration index (*Z*_*p*_), which has been widely demonstrated to be a reliable indicator of the degree of cell membrane permeabilization induced by PEF treatment in diverse agri-food by-products tissues, including those derived from winemaking process (2, 19, 21, 38).

Based on the experimental design (FC-CCD), Table 1 shows the influence of the input variables, namely electric field strength and energy input, on the *Z*_*p*_ value of PEF-treated grape pomace tissues. Results reveal that the extent of cell membrane permeabilization significantly (*p* ≤ 0.05) increased with increasing the field strength and energy input, with the difference being not significant (p ≤ 0.05) only when the field strength was changed from 2.75 to 5 kV/cm at a fixed energy input of 10.5 kJ/kg. Moreover, in the range of the investigated PEF treatment conditions, the effect of field strength appeared slightly more pronounced than that of the energy input. The highest *Z*_*p*_ value was detected when the most intense PEF treatment was applied (5 kV/cm and 20 kJ/kg).

The increment of *Z*_*p*_ values with increasing the intensity of PEF treatment observed in this study is consistent with previously reported findings for different plant tissues, including grape pomace (2, 21, 38). As an example, in the study of Carpentieri et al. (2), who investigated the optimization of PEF-assisted extraction of phenolic compounds from white grape pomace, the electric field strength applied showed a remarkable influence on the Z_*p*_ value, while the effect of energy input appeared more evident especially at lower field strengths. In particular, the highest value of the cell disintegration index (0.80) was attained when applying an electric field strength of 5 kV/cm and an energy input of 10.5 kJ/kg. The apparent higher resistance to the electropermeabilization treatment exhibited by the red grape pomace tissues in the present work might be ascribed to their higher content in insoluble dietary fibers (cellulose, hemicellulose, lignin), as compared to the white grape pomace (9), which, therefore, might hinder the electroporation of the cell envelope of red grape pomace tissue. Moreover, it should also be taken into account that, as compared with white grape pomace, besides the crushing step and prior to the pressing phase, red grape usually undergo a long maceration process (up to days), during which the grape tissues could further lose their structural integrity, thus making the PEF treatment less effective in permeabilizing the cell tissues of the resulting pomace.

### 3.2. Model fitting and optimization of PEF processing conditions

A second-order polynomial equation (Equation 2) was selected to fit the data obtained from the experimental design (FC-CCD). The values and significance of the regression coefficients of the predicted polynomial model are reported in Table 4. Results show that the linear terms of both factors (field strength and energy input) exerted a highly significant effect (*p* ≤ 0.001) on the permeabilization degree of the plant tissue, while interactions between the single factors were not significant (*p* > 0.05). However, in agreement with the findings previously achieved in the case of white grape pomace (2), the electric field strength appeared as the factor that exhibited the most pronounced effect on the response variable, showing a significant effect on *Z*_*p*_, with respect to the non-significant (*p* > 0.05) quadratic term of the energy input. In addition, the significant negative value of the quadratic coefficient (β_11_) suggests that the field strength can achieve an optimum value that maximizes the response variable.

**Table 4:** Analysis of variance (ANOVA) of the second order polynomial equation describing the influence of PEF process parameters on the cell disintegration index (*Z*_*p*_) of grape pomace tissues.

**Coefficients**	**Z**_***p***_	
β_0_	−0.148433	
β_1_ (E)	0.240292	***
β_2_ (W_T_)	0.018985	***
β_12_ (E x W_T_)	−0.002172	ns
β_11_ (E x E)	−0.028986	**
β _22_ (W_T_ x W_T_)	−0.000562	ns
p value of the model	0.0001	***
R^2^	0.9545	
RMSE	0.3504	

ns, not significant for p > 0.05. ^**^Significant for p ≤ 0.01; ^***^Significant for p ≤ 0.001. RMSE, Root Mean Square Error.

Table 4 also reports the results of the ANOVA for the significant terms of the selected second-order polynomial model and the statistics used to test its adequacy. The *p*-value of the model suggested that it was significant (*p* < 0.0001) for the selected response, thus corroborating the effectiveness of the model to describe the experimental data. In addition, the Root Mean Square Error (RMSE = 0.3504) and the determination coefficient (R^2^ = 0.9545) values indicated a good correlation between the experimental data and the predicted values.

**Figure 1:**
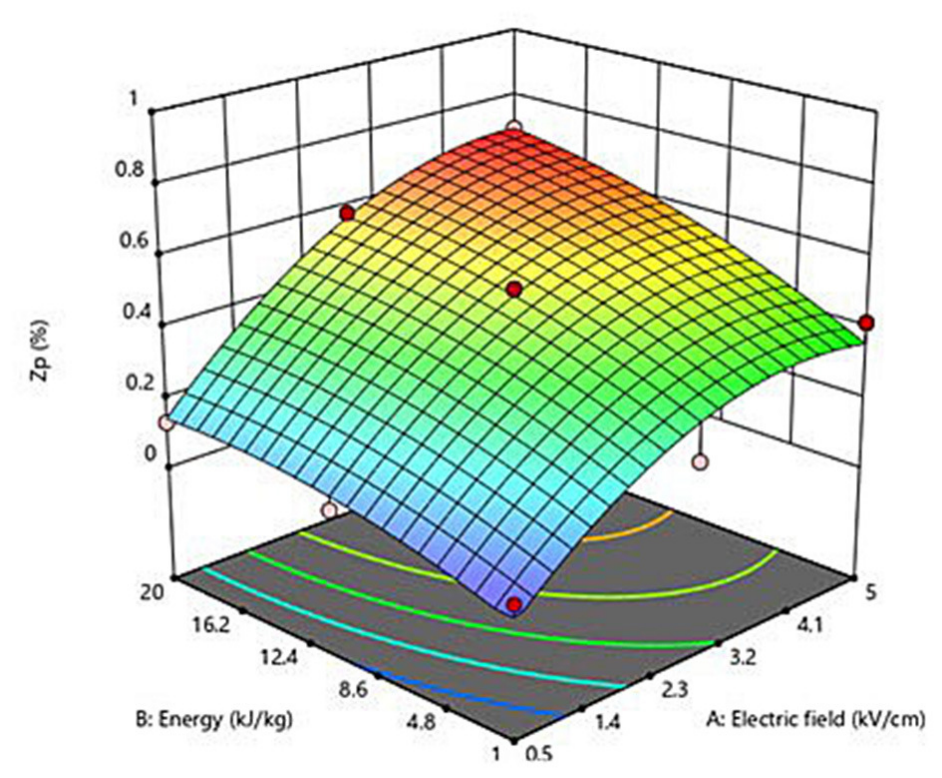
Response surface plot showing the influence of electric field strength (kV/cm) and energy input (kJ/kg) on the cell disintegration index (*Z*_*p*_) of grape pomace tissues.

Figure 1 depicts the three-dimensional response surface graph that shows the interactions between field strength and energy input and their effect on the *Z*_*p*_ of PEF-treated grape pomace tissues. As previously discussed, the increase in the PEF treatment intensity led to an increase in the degree of cell membrane permeabilization of grape pomace tissues. In particular, in accordance with the coefficients and significance of each factor involved in the model, the graph clearly demonstrates that *Z*_*p*_ increased almost linearly with increasing the energy input, whereas the field strength mainly affected the observed response in a quadratic way. These results confirm the effectiveness of PEF treatment to induce the cell membrane electroporation of red grape pomace tissues in a mostly field strength-dependent way. Moreover, the obtained results allowed to define the optimal PEF treatment conditions as the minimal electric field strength (*E*_*opt*_, kV/cm) and total specific energy input (*W*__*T*_, *opt*_, kJ/kg) that led to the highest degree of cell membrane permeabilization. Specifically, the maximum *Z*_*p*_ value (0.70) was observed for the PEF-treated samples at 4.6 kV/cm and 20 kJ/kg. These optimal conditions were used to investigate the effect of PEF pre-treatment application on the extractability of bioactive compounds from red grape pomace.

### 3.3. Effect of PEF-assisted extraction on the recovery of bioactive compounds from red grape pomace

#### 3.3.1. Model fitting

Several variables, namely diffusion time, temperature, type of solvent, and solid/liquid ratio, have been shown to significantly influence the recovery yields of valuable intracellular compounds from winemaking residues (25–27, 30, 39).

In the present study, the FC-CCD was constructed to systematically investigate the effect of three independent factors, namely ethanol concentration, extraction time, and temperature, on total phenolic content (TPC), flavonoid content (FC), total anthocyanin content (TAC), tannin content (TC), and antioxidant activity (FRAP) of red grape pomace extracts, achieved from both conventional SLE and PEF-assisted extraction process (Tables 2, 3).

Overall, the obtained results demonstrate that the three independent factors considerably affected all the investigated response variables. Specifically, regardless of the application of PEF pre-treatment, the maximum levels of the responses were attained at the highest ethanol concentration (50%, v/v), extraction time (300 min), and temperature (50°C) investigated. Moreover, results also show that the permeabilization effect induced upon the application of PEF pre-treatment to red grape pomace at the selected optimal conditions (4.6 kV/cm – 20 kJ/kg), significantly intensified the extractability of TPC, FC, TAC, and TC, and led to higher antioxidant activity of the extracts, with respect to the conventional SLE.

To quantify the influence of the three investigated factors on the recovery of bioactive compounds and antioxidant activity of the extracts of either untreated and PEF-treated grape pomace, a two-factor interaction (2FI) model (Equation 3) was used to fit the experimental data obtained from the FC-CCD for all the investigated response variables (Tables 2, 3).

The values and significance of the regression coefficients of the polynomial models and corresponding p values, the determination coefficient (R^2^), and the RSME for each variable are reported in Tables 5, 6.

**Table 5:** Analysis of variance (ANOVA) of the two-factor interaction (2FI) model describing the influence of the SLE extraction parameters on the TPC, FC, and antioxidant activity (FRAP) of untreated and PEF (4.6 kV/cm, 20 kJ/kg)-treated grape pomace.

**Coefficients**	**SLE**						**PEF-assisted extraction**					
	**TPC**		**FC**		**FRAP**		**TPC**		**FC**		**FRAP**	
	**(mgGAE/g**_DM_ **)**		**(mgQE/g**_DM_ **)**		**(mgAAE/g**_DM_ **)**		**(mgGAE/g**_DM_ **)**		**(mgQE/g**_DM_ **)**		**(mgAAE/g**_DM_ **)**	
β_0_	0.71989		2.80043		1.30268		0.54185		10.94041		1.77311	
β_1_ (T)	−0.020805	***	−0.000852	***	−0.018266	**	−0.012244	***	−0.184748	***	−0.027565	**
β_2_ (time)	−0.007851	**	−0.028664	***	−0.006359	*	−0.005244	**	−0.036614	***	−0.008054	*
β_3_ (Ethanol)	−0.026822	***	−0.095284	***	−0.040341	**	−0.046563	***	−0.467007	***	−0.060277	**
β_12_ (T x t)	0.000284	*	0.001473	*	0.000196	*	0.000210	*	0.001972	ns	0.000235	*
β_13_ (T x Ethanol)	0.002194	**	0.006593	*	0.001238	*	0.003348	***	0.016873	**	0.001802	**
β_23_ (t x Ethanol)	0.000159	*	0.000693	*	0.000113	*	0.000120	*	0.001093	ns	0.000156	*
p value of the model	<0.0001	***	0.0010	***	0.0062	**	<0.0001	***	0.0004	***	0.0090	**
R^2^	0.953		0.903		0.848		0.980		0.916		0.833	
RMSE	0.003		0.905		0.011		0.016		0.622		0.006	

ns, not significant for p > 0.05. ^*^Significant for p ≤ 0.05; ^**^Significant for p ≤ 0.01; ^***^significant for p ≤ 0.001. RMSE, Root Mean Square Error.

**Table 6:** Analysis of variance (ANOVA) of the two-factor interaction (2FI) model describing the influence of the SLE extraction parameters on the TAC and TC of untreated and PEF (4.6 kV/cm, 20 kJ/kg)-treated grape pomace.

**Coefficients**	**SLE**				**PEF-assisted extraction**			
	**TAC**		**TC**		**TAC**		**TC**	
	**(mgC3G/g**_DM_ **)**		**(mgTC/g**_DM_ **)**		**(mgC3G/g**_DM_ **)**		**(mgTC/g**_DM_ **)**	
β_0_	0.102999		−1.32124		0.127020		0.832636	
β_1_ (T)	−0.002208	ns	0.022951	***	−0.003415	**	−0.029764	ns
β_2_ (time)	−0.000952	ns	0.002893	***	−0.000845	*	−0.006591	ns
β_3_ (Ethanol)	0.000054	***	0.006375	***	−0.001365	***	−0.018340	**
β_12_ (T x t)	0.000027	ns	−6.87·10^−6^	ns	0.000026	ns	0.000264	ns
β_13_ (T x Ethanol)	0.000129	ns	0.000799	**	0.000238	*	0.001478	ns
β_23_ (t x Ethanol)	0.000028	ns	0.000047	ns	0.000025	*	0.000099	ns
p value of the model	0.0100	**	<0.0001	***	0.0003	***	0.0410	*
R^2^	0.815		0.970		0.929		0.743	
RMSE	0.028		0.705		0.120		0.469	

ns, not significant for p > 0.05. ^*^Significant for p ≤ 0.05; ^**^Significant for p ≤ 0.01; ^***^Significant for p ≤ 0.001. RMSE, Root Mean Square Error.

Results show that, for the extracts of either untreated or PEF-treated grape pomace, all the investigated factors resulted in a statistically significant linear effect on TPC, FC, and FRAP values, with a non-significant effect detected only for the linear terms of temperature and diffusion time for both TAC of untreated samples and TC of PEF-treated samples. Similarly, regardless of the PEF pre-treatment, all the interactions between single factors were significant for TPC and FRAP. In the case of FC, instead, all the interactions were significant for the control samples, while only the dependence of extraction temperature and ethanol concentration was detected in the case of PEF-treated samples. This implies that, in the investigated variable domain, PEF-pre-treatment only amplifies the interaction of extraction temperature and ethanol concentration on the extractability of flavonoids, while decreasing the effect of temperature on diffusion time and that of diffusion time on ethanol concentration.

Regarding TAC, all the interactions between single factors were not significant for the control extraction, whereas it was detected that only the extraction temperature exerted a not significant dependence on the diffusion time for the PEF-treated samples. This implies that, in the investigated variables domain, PEF pre-treatment decreased the effect of extraction temperature on diffusion time, with respect to the untreated samples.

Finally, with respect to TC, only the temperature-ethanol concentration interaction for the untreated samples was found to be significant.

These results are partially in agreement with those found by Carpentieri et al. (2), who found that all the investigated factors (ethanol concentration, temperature and time) turned out in a statistically significant linear effect on the majority of the response variables (TPC, FC, and FRAP), with the exception of the linear term of ethanol concentration in the case of FRAP values of PEF treated samples. Furthermore, they also found that all the interactions between single factors were not significant for PEF-treated samples, while only the dependency of the extraction temperature on the diffusion time for TPC, FC and FRAP and that of temperature and ethanol concentration for FRAP was detected in the case of untreated samples. It should also be worth highlighting that, independently of the response variables and extraction method, the non-significance of the quadratic terms detected in the present work, which therefore were not considered for the general evaluation of the model, means that for red grape pomace extracts no maximum response value can be expected within the investigated variables domain. This is partially in contrast with results achieved for white grape pomace extracts (2), where, likely due to the larger range of ethanol concentration investigated (0–100%), a negative quadratic term of the ethanol concentration was observed.

The ANOVA (Tables 5, 6) showed that the RSME values were lower than 0.905, and that the relationship between response variables and the extraction parameters had determination coefficient (R^2^) values ranged between 0.743 and 0.980, which indicates a good correlation between the experimental data and those predicted by the model.

Additionally, analysis of variance indicated that the model used was significant (*p* ≤ 0.04) for all the responses, thus supporting the predictive efficacy of the selected model.

#### 3.3.2. RSM analysis and optimization of the extraction processing conditions

**Figure 2:**
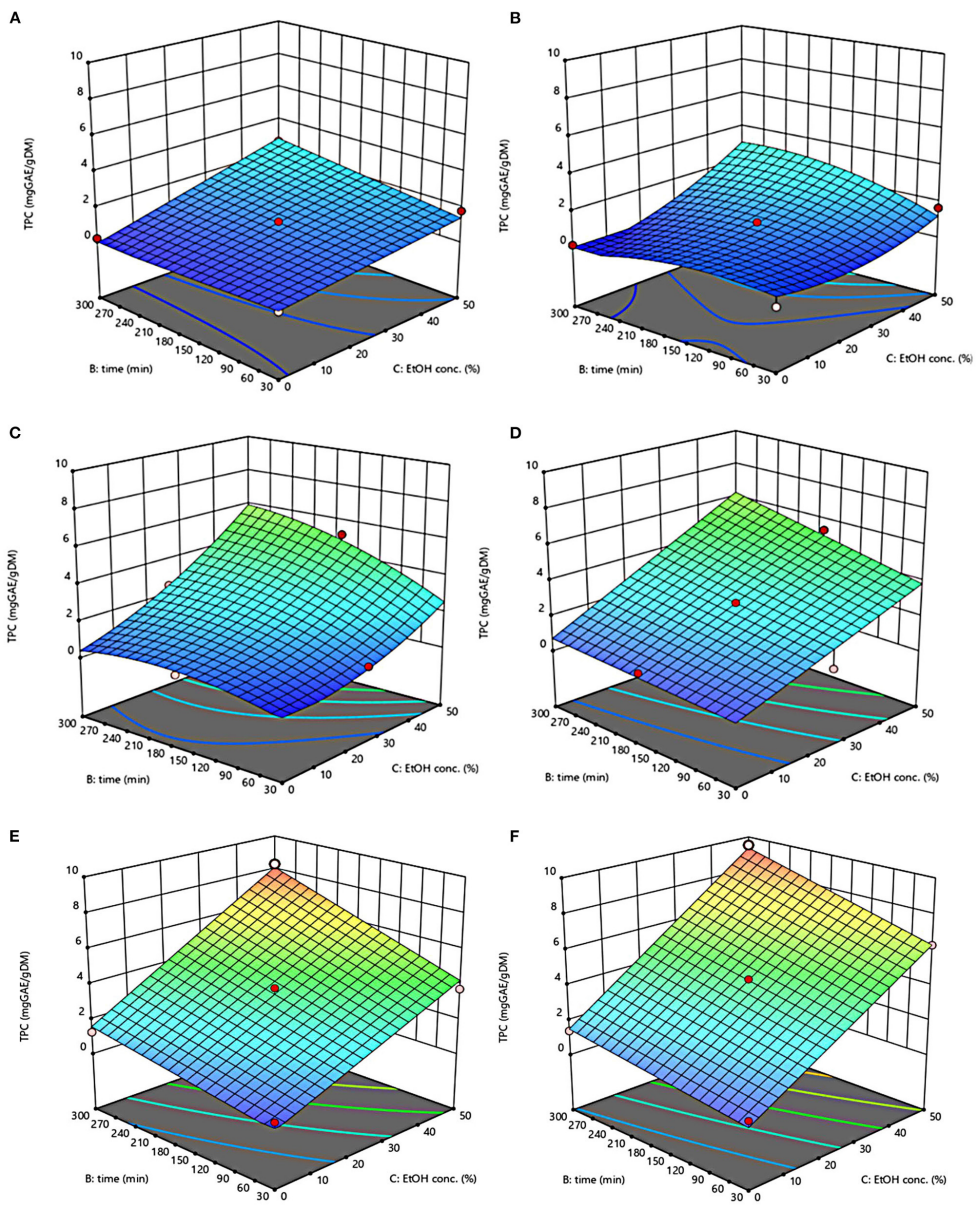
Response surfaces of Total Phenolic Content (TPC) of extracts from untreated (Control) **(A, C, E)** and PEF-treated (E = 4.6 kV/cm; WT = 20 kJ/kg) **(B, D, F)** grape pomace as a function of extraction time and ethanol concentration-Extraction temperature set at 20°C **(A, B)**, 35°C **(C, D)**, 50°C **(E, F)**.

**Figure 3:**
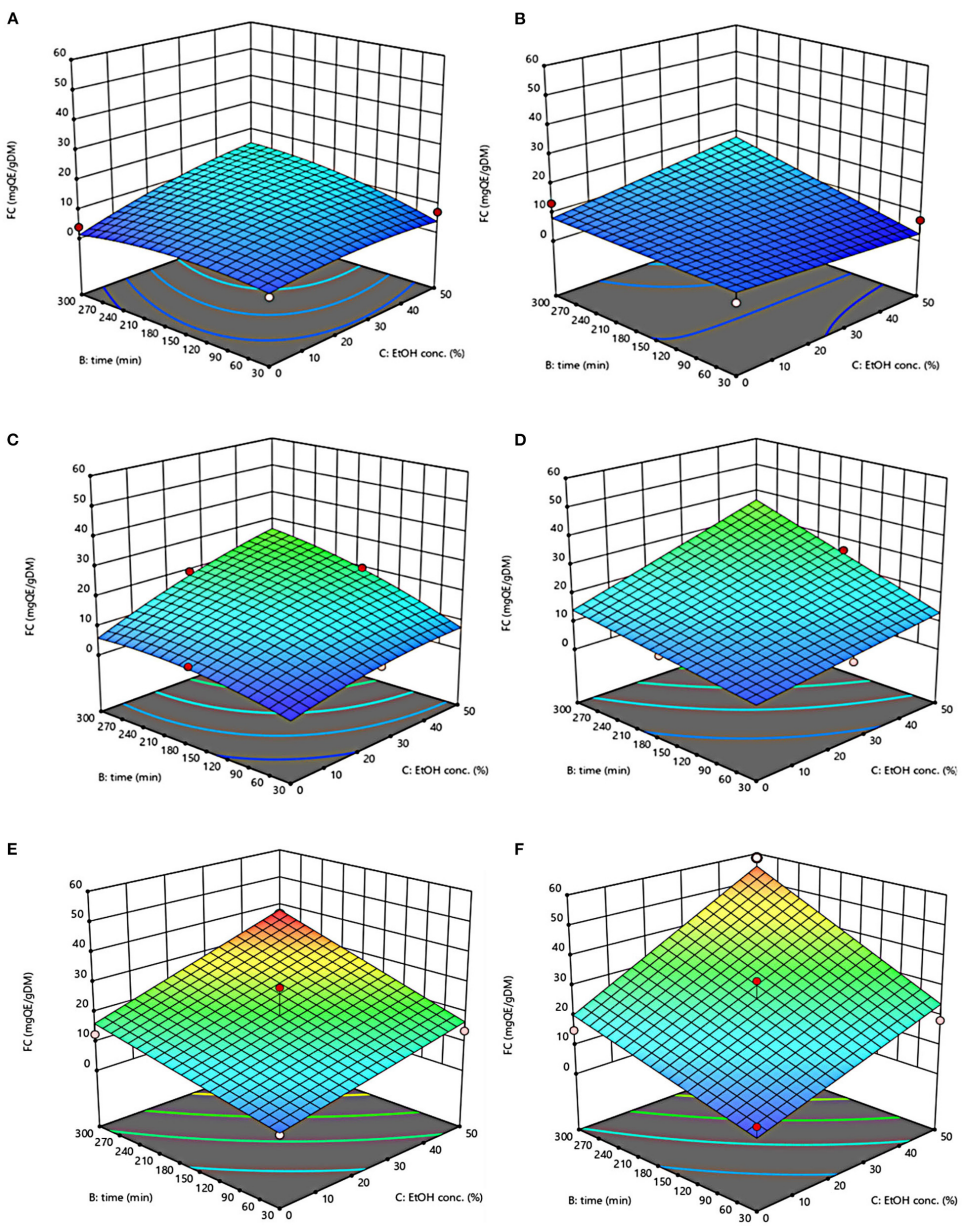
Response surfaces of Flavonoid Content (FC) of extracts from untreated (Control) **(A, C, E)** and PEF-treated (E = 4.6 kV/cm; WT = 20 kJ/kg) **(B, D, F)** grape pomace as a function of extraction time and ethanol concentration. Extraction temperature set at 20°C **(A, B)**, 35°C **(C, D)**, 50°C **(E, F)**.

**Figure 4:**
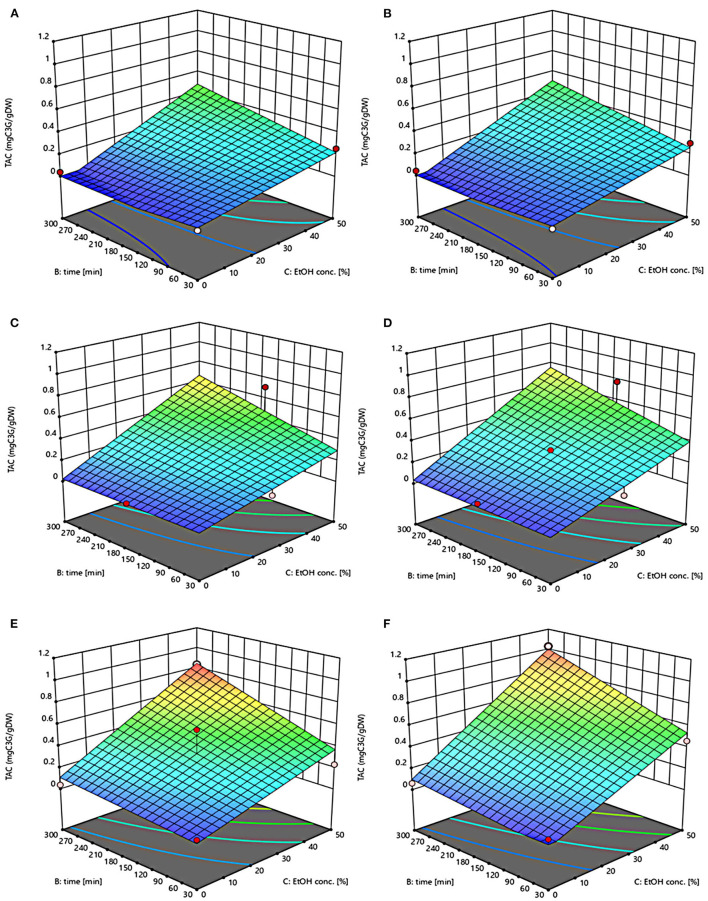
Response surfaces of Total Anthocyanin Content (TAC) of extracts from untreated (Control) **(A, C, E)** and PEF-treated (E = 4.6 kV/cm; WT = 20 kJ/kg) **(B, D, F)** grape pomace as a function of extraction time and ethanol concentration. Extraction temperature set at 20°C **(A, B)**, 35°C **(C, D)**, 50°C **(E, F)**.

**Figure 5:**
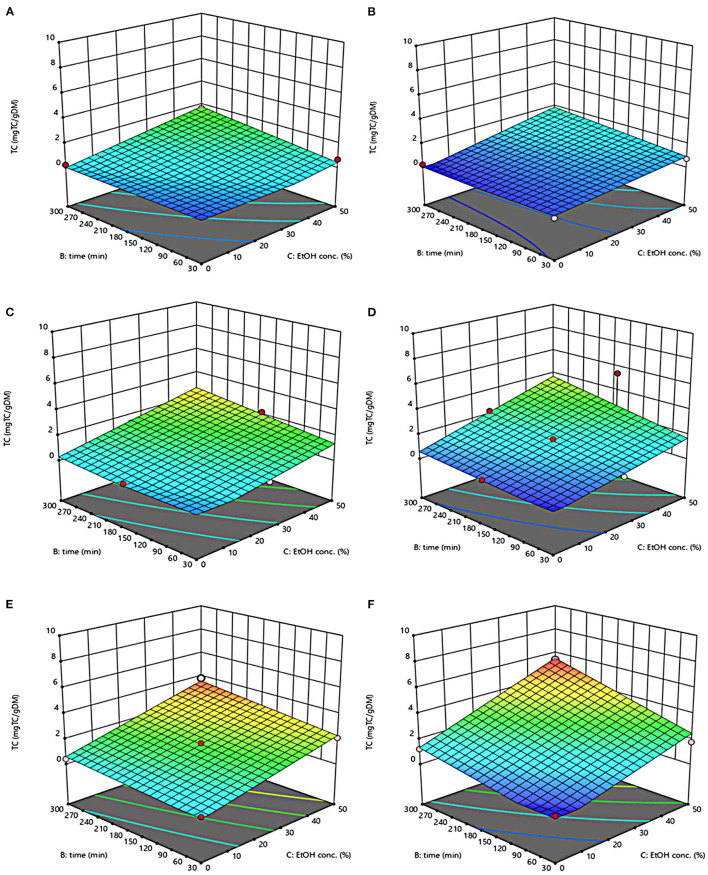
Response surfaces of Tannin Content (TC) of extracts from untreated (Control) **(A, C, E)** and PEF-treated (E = 4.6 kV/cm; WT = 20 kJ/kg) **(B, D, F)** grape pomace as a function of extraction time and ethanol concentration. Extraction temperature set at 20°C **(A, B)**, 35°C **(C, D)**, 50°C **(E, F)**.

The three-dimensional response surface plots reported in Figures 2–5 depict the interaction of the extraction temperature (20–50°C), diffusion time (30–300 min), and ethanol concentration (0–50%, v/v) on the level of TPC, FC, TAC, and TC of the extracts from untreated and PEF-treated grape pomace. It can be seen that, within the whole investigated domain, the behavior of all the response variables appeared similar, which is consistent with previous findings (40, 41). Moreover, the application of PEF pre-treatment to grape pomace increased the amount of extracted bioactive compounds, as compared with control extraction.

Even though all the investigated variables had a statistically significant (*p* ≤ 0.041) effect on the extraction yield of the target compounds, the ethanol concentration in water and the extraction temperature appeared as the factors that most influenced the observed responses. This is also confirmed by the higher value of the linear coefficient of the ethanol concentration and temperature than that of diffusion time (Tables 5, 6). Moreover, regardless of the application of PEF treatment, the influence of ethanol concentration and especially that of diffusion time on the extraction yield of bioactive compounds appeared more pronounced only at extraction temperature higher than 35°C.

The positive effect of moderate extraction temperature on the extractability of valuable compounds from untreated or PEF-treated grape by-products was previously observed by other scientists (2, 25, 26, 42). It can be explained considering that higher temperature improves the mass transfer efficiency by enhancing the solubility and diffusivity of intracellular compounds in the solvent while reducing its surface tension and viscosity (25, 26). On the other hand, mild heating of plant tissues may contribute to enhancing membrane permeability by affecting lipid bilayer composition and interactions between lipids and membrane proteins (43). Additionally, an increase in temperature may induce the disruption of hydrogen bonds between the cell wall and phenolic compounds, thus intensifying their extractability and diffusivity in the solvent (44). Nonetheless, there are practical constraints limiting the increment of temperature, which are related, among others, to the fact that the exposure of plant tissues of grape residues to temperatures above 50–60°C might trigger the thermal degradation of colored phenolic compounds (25, 29, 42).

Regarding the influence of ethanol concentration on the observed responses, it can be attributed to the fact that ethanol concentration affects the polarity of the solvent mixture, thus allowing the recovery of phenolic compounds with a broad spectrum of polarities (32). In addition, it is known that ethanol may alter the barrier properties of the plant cell by acting on the phospholipids bilayer of the cytoplasmic membrane, thus facilitating the capacity of penetration of the solvent into the cell and the subsequent mass transfer of the solubilized compounds (45).

The key role played by the ethanol concentration and temperature on the extractability of phenolic compounds from winemaking residues was previously investigated by other researchers (26, 29, 30). For example, in agreement with our findings, Rajha et al. (25) and Carpentieri et al. (2) found that the TPC and FC values reached their peak at 50°C during the SLE of phenolic compounds. Carpentieri et al. (2) and Caldas et al. (27) found that an increase in ethanol concentration induced an increment in the TPC up to reach a maximum value at 50%, which is the highest value tested in this work, while further increment of ethanol concentration reduced the amount of phenolic compound in the obtained extracts. Similar results were also observed by other scientists when investigating the effect of ethanol concentration on the recovery of phenolic compounds from food by-products different from grape pomace, such as potato peels (32) and medicinal plant (46).

On the other hand, ethanol-water mixtures have been shown to facilitate the extractability and release of anthocyanins into the solvent (47). In this regards, Monrad et al. (48) found that 50% (v/v) ethanol-water mixture extracted the highest amount of total procyanidins from dried red grape pomace (48), which is consistent with our findings. Similarly, other authors found that acidified 50% ethanol-water mixture achieved the highest anthocyanin extractability from either grape marc (49) or blueberry press cake (50).

Moreover, the results of Figure 5 highlight the positive effect of water-ethanol solvent on the extractability of tannins. They can be explained considering that the solubility of tannins is highly affected by the presence of ethanol in the solvent, which leads to a reorganization of the lipids, thus favoring the solubilization and release of intracellular tannins from grape seeds and skins (47).

Regarding the extraction time, results revealed that regardless of the application of PEF pre-treatment, TPC, FC, TAC, and TC value were scarcely affected by the diffusion time at lower temperature (20–35°C), while slightly increased with increasing the diffusion time at the highest temperature tested (50°C), with the highest level of bioactive compounds being detected after 300 min of extraction. This is partially in contrast with findings previously reported by other scientists, who found a quadratic significant negative effect of diffusion time on the extraction yield of phenolic compounds, showing a maximum value at an intermediated time within the investigated range (2, 25, 30). For example, Carpentieri et al. (2) found a slight increase of TPC and FC in white grape pomace extracts with increasing the diffusion time (30–300 min), but only at a temperature lower than 35°C. At higher temperature (50°C), instead, the same authors found that the maximum level of TPC and FC was reached at an intermediate extraction time (190–223 min), depending on the extraction method and response variable, while a prolonged extraction time resulted in a decrease of TPC and FC values, likely due to the occurrence of oxidation reactions or degradation phenomena, which would be accelerating at higher temperature (25). This different behavior could be in part attributed to the different proximate composition of white and red grape pomace, with the latter being richer in lignocellulosic compounds. The presence of these compounds might hinder the unlocking and release of intracellular compounds especially at room temperature even after exposure to long extraction time, thus requiring higher temperatures able to weaken the cell envelope and consequently improve the mass transfer efficiency (9). On the other hand, these different results could be also partially attributed to the fact that, the red grape pomace, unlike the white one, was generated from a different winemaking process, which included a long (10 days) maceration time during which it is likely that a large amount of precious intracellular compounds, especially those not strongly bounded to the cellular structure, were released. This probably made the recovery of the remaining amount of bioactive compounds from red grape pomace more difficult during the subsequent conventional and PEF-assisted extraction processes. Therefore, further studies should be carried under more severe processing conditions than those tested in this study, in order to verify this hypothesis and achieve higher extraction yield.

The optimal values of the three independent factors that maximize all the investigated response variables were shown by the adopted model to be 50°C, 50% ethanol-water mixture, and 300 min for extracts from both untreated and PEF-treated grape pomace. Specifically, under these optimal conditions the TPC, FC, TAC, and TC values were 8.30 mgGAE/g_DM_, 36.68 mgQE/g_DM_, 0.84 mgC3G/g_DM_, and 3.84 mgTC/g_DM_, respectively, for the control sample, and 9.51 mgGAE/g_DM_, 58.53 mgQE/g_DM_, 1.03 mgC3G/g_DM_, and 5.45 mgTC/g_DM_, respectively, for the PEF-treated samples. Therefore, the application of optimized PEF-assisted extraction process can be successfully used to intensify the extractability of phenolic compounds (15%), flavonoids (60%), anthocyanins (23%), and tannins (42%). According to data reported in Table 1, this can be ascribed to the electroporation effect induced by PEF treatment (*Z*_*p*_ = 0.70), which facilitates the penetration of the solvent into the cytoplasm of the plant cell and the subsequent mass transfer of the solubilized intracellular compounds, thus enhancing the extractability of target compounds. Furthermore, it is important to note that the ability of ethanol and moderate temperature to further affect the barrier properties of the cell membrane of the plant tissues, as well as to improve the solubility and diffusivity of the intracellular compounds in the solvent may have contributed in ameliorating the extractability of the target compounds.

Even though any comparison with the extraction yields of bioactive compounds from winemaking residues previously reported in literature is very tough and challenging being dependent on several factors (grape variety, ripening conditions, type of by-product, equipment and experimental protocols), the results obtained in this work are somehow consistent with those reported in current literature. In particular, the optimal extraction conditions determined in the present work in terms of temperature (50°C) and ethanol concentration (50%) are the same found by other scientist (25, 27) during the SLE of phenolic compounds from red grape by-products. Moreover, the optimal concentration of phenolic compounds, flavonoids, anthocyanins, and tannins achieved in this work were consistent with TPC (2.4–6.1 mg/g_DW_), FC (21.46 mg/g_DW_), TAC (0.84–1.31 mg/g_DW_) and TC (3–110 mg/g_DW_) of extracts from grape pomace reported in previous studies (25, 26, 51, 52).

**Figure 6:**
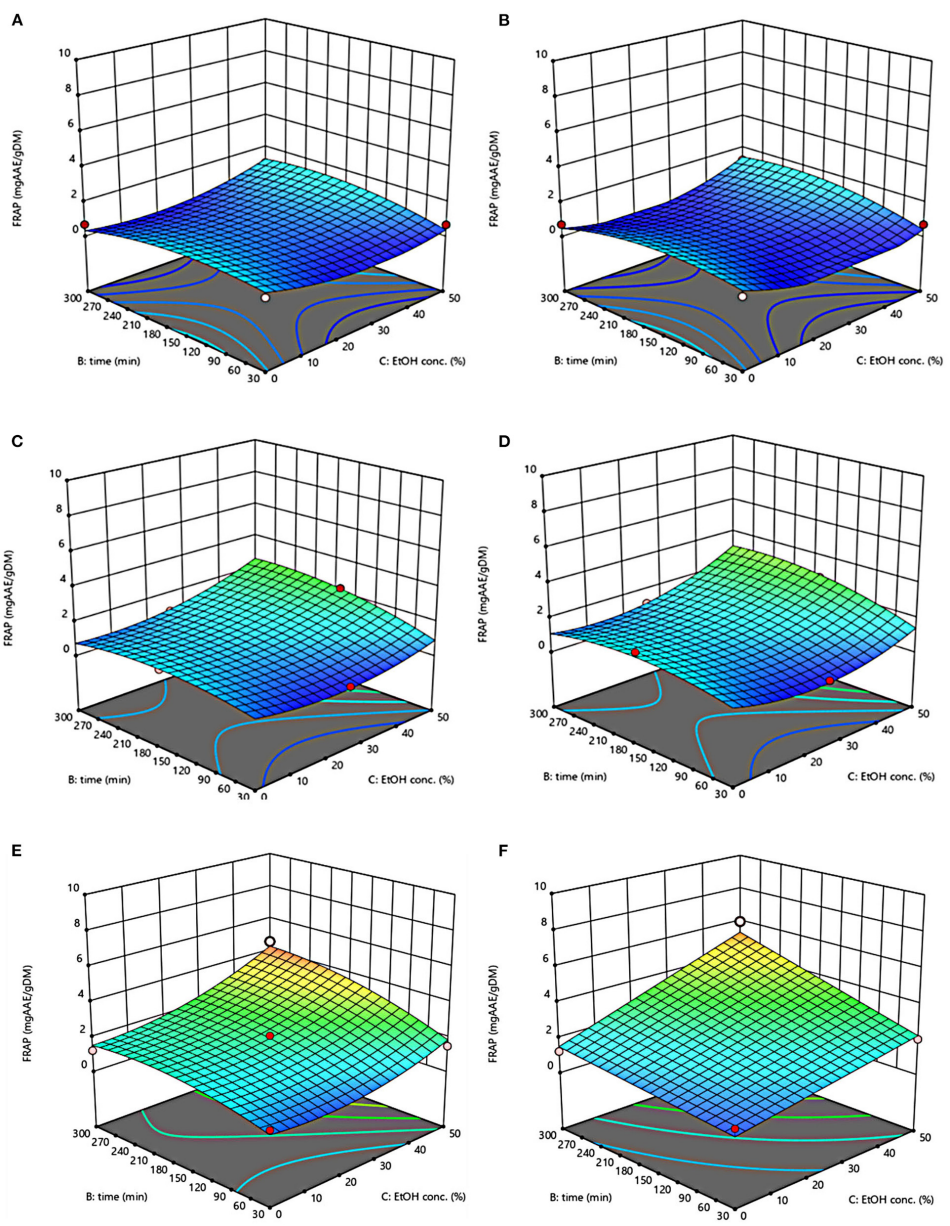
Response surfaces of antioxidant activity (FRAP) of extracts from untreated (Control) **(A, C, E)** and PEF-treated (E = 4.6 kV/cm; WT = 20 kJ/kg) **(B, D, F)** grape pomace as a function of extraction time and ethanol concentration. Extraction temperature set at 20°C **(A, B)**, 35°C **(C, D)**, 50°C **(E, F)**.

Response surfaces of antioxidant activity (FRAP) of extracts from untreated and PEF-treated grape pomace are depicted in Figure 6. Results reveal that regardless of the application of PEF pre-treatment, the ethanol-water concentration and the extraction temperature appeared as the factors that most affected the FRAP values, thus reflecting the trend observed for TPC, FC, TAC, and TC. On the other hand, in comparison with control extracts, PEF-treated samples exhibited higher antioxidant activity, especially at a higher temperature, likely due to the high amount of phenolic compounds recovered upon the electropermeabilization treatment. Moreover, a strong positive correlation was observed between the TPC, FC, TAC, FC and FRAP values, with a Pearson correlation coefficient in the range 0.88–0.89 for TPC, 0.86–0.92 for FC, 0.78–0.84 for TAC, and 0.82–0.89 TC, suggesting that phenolic compounds mostly contribute to the global antioxidant activity of the grape pomace extracts, as previously observed in previous literature works (2, 24).

The values of the factors that maximize antioxidant activity were the same found for the other response variables, namely 50°C, 50% ethanol-water mixture, and 300 min for extracts from both untreated and PEF-treated grape pomace, resulting in antioxidant activity of 4.58 mgAAE/g_DM_ and 5.99 mgAAE/g_DM_, respectively. These results appear somehow consistent with those observed by Melo et al. (26), who found that the highest level of antioxidant activity of extracts achieved from grape pomaces of different grape varieties, was obtained at moderate ethanol concentrations (40–60%, v/v) and higher temperature.

Based on the results reported so far, further experiments aimed at investigating the effect of PEF pre-treatment on the phenolic composition of the extracts from red grape pomace were carried out with the PEF (4.6 kV/cm and 20 kJ/kg) and SLE (50°C, 50% ethanol-water mixture, 300 min) set at their optimal conditions.

#### 3.3.3. Quantification of the main phenolic compounds via HPLC-PDA analysis

**Figure 7:**
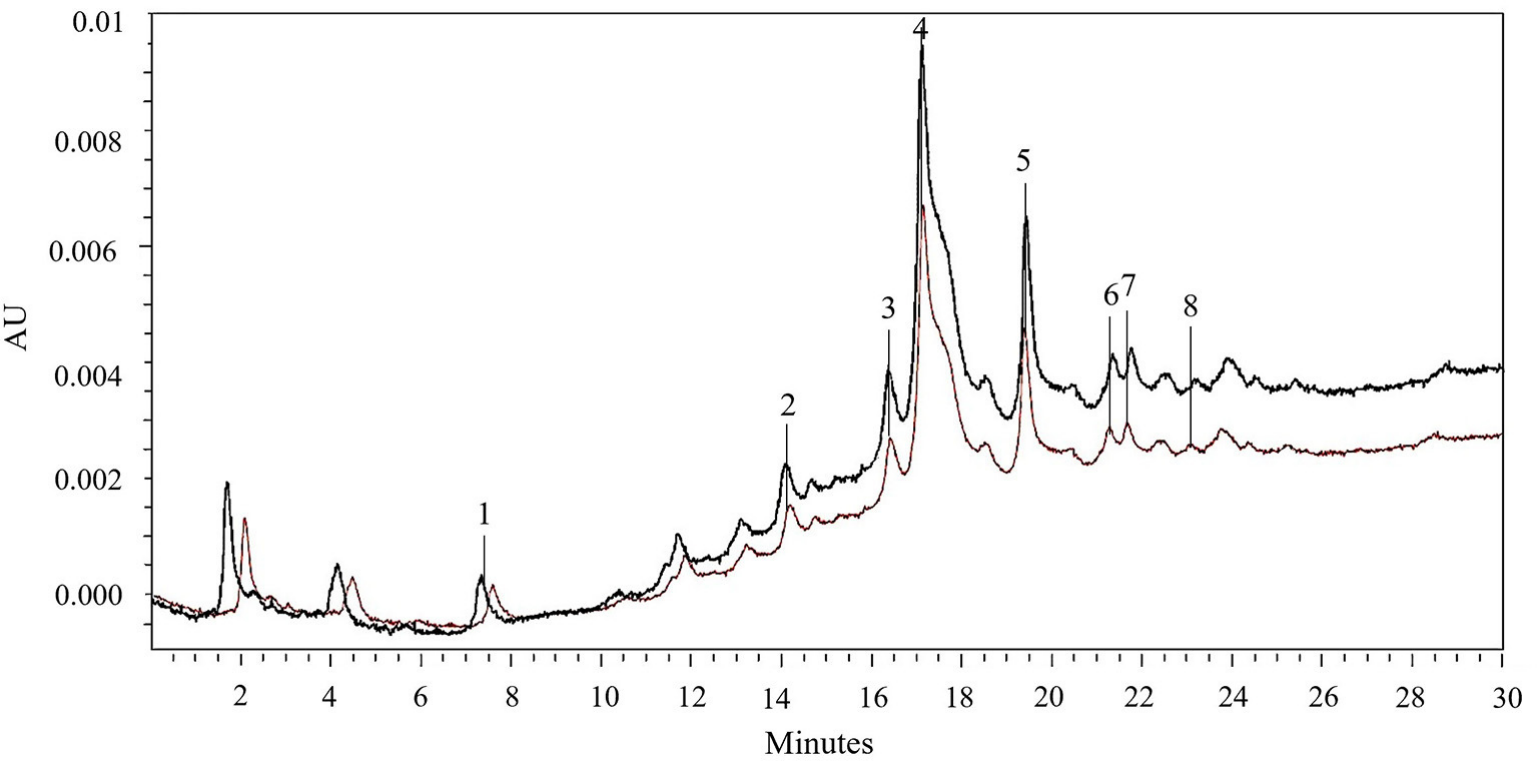
HPLC-PDA chromatograms of 50% (v/v) ethanol-water extracts obtained after 300 min of extraction at 50°C from untreated (brown line) and PEF (E_opt_ = 4.6 kV/cm; W_T,opt_ = 20 kJ/kg)-treated (black line) red grape pomace. Peak identification: gallic acid (1); chlorogenic acid (2); caffeic acid (3); epicatechin (4); p-coumaric acid (5); naringin (6); rutin (7); phlorizin (8).

The identification and quantification of the main phenolic compounds in the 50% ethanol-water extracts obtained from untreated and PEF(E_opt_ =4.6 kV/cm; W_T,opt_ = 20 kJ/kg)-treated red grape pomace after 300 min SLE at 50°C, was assessed *via* HPLC-PDA analysis. The resulting chromatogram profiles and the concentrations of the identified phenolic compounds are presented in Figure 7 and Table 7, respectively.

**Table 7:** Concentrations (mg/g_DW_) of gallic acid, chlorogenic acid, caffeic acid, epicatechin, p-coumaric acid, naringin, rutin and phlorizin (HPLC/PDA analysis) in the extracts from untreated and PEF-treated grape pomace.

**Peak no**.	**Compound**	**Max absorption wavelength (nm)**	**Retention time (min)**	**Concentration (mg/g**_**DW**_ **)**	
				**Untreated**	**PEF-treated**
1	Gallic acid	271	7.60	0.074 ± 0.01a	0.083 ± 0.01b
2	Chlorogenic acid	320	14.76	0.20 ± 0.01a	0.27 ± 0.02b
3	Caffeic acid	320	16.42	0.099 ± 0.01a	0.12 ± 0.01a
4	Epicatechin	280	17.15	2.99 ± 0.03a	4.83 ± 0.05b
5	p-coumaric acid	320	19.40	0.38 ± 0.02a	0.46 ± 0.03b
6	Naringin	283	21.28	0.25 ± 0.01a	0.27 ± 0.02a
7	Rutin	260	21.68	0.26 ± 0.02a	0.29 ± 0.03a
8	Phlorizin	280	23.12	0.059 ± 0.01a	0.063 ± 0.01a

Data are expressed as means (n = 3) ± SDs. Values with different lowercase letters within the same row are significantly different (p ≤ 0.05).

Results reveal that regardless of the application of PEF pre-treatment, epicatechin (peak 4) was the most abundant phenolic compound detected in the extracts, followed by p-coumaric acid (peak 5), rutin (peak 7), naringin (peak 6), chlorogenic acid (peak 2), and to a lesser extent by caffeic acid (peak 3), gallic acid (peak 1), and phlorizin (peak 8). These results are consistent with the fact that epicatechin is the most common flavonoid found in grapes (53), which possess high antioxidant power and health-beneficial effects against chronic diseases (54). Additionally, as can be seen, the HPLC chromatogram profiles of the extracts from untreated and PEF-treated samples appeared to be similar (Figure 7), which is consistent with the findings reported by other scientists when analyzing phenolic extracts achieved from different agri-food by-products, including those derived from winemaking process (2, 21, 32, 50, 55). This is probably due to the relatively low intensity of the applied electrical treatment, which neither induced the selective extraction of specific compounds nor triggered degradation reactions.

However, it is worth noting that, in comparison with the control samples, PEF pre-treatment increased the peak area of all phenolic compounds. In particular, coherently with the results of Figures 2–5, the application of PEF pre-treatment caused a significant increment in the concentration of gallic acid (by 12%), chlorogenic acid (by 35%), epicatechin (by 62%), and p-coumaric acid (by 21%), whereas the increase of the other phenolic compounds was not statistically significant.

Results reported in Table 7 appear somehow consistent with those found by other authors. For example, Antoniolli et al. (56) and Monrad et al. (48) bioactive compounds from red grape pomace and found that the epicatechin content in the 50% ethanol-water extracts ranged between 1.76 and 5.53 mg/g_DW_ (48, 56). On the other hand, Brianceau et al. (21) detected the presence of epicatechin in the extracts from PEF-treated grape pomace, even though no statistically significant differences were observed with respect to the extracts from untreated sample.

Grape pomace, and especially grape skins, is also a rich source of anthocyanins. Among them, 3-O-glucosides of peonidin, malvidin, petunidin, cyanidin, and delphinidin are reported to be the most abundant anthocyanins in grape skin extracts, with differences in their composition having been detected depending on several factors such as grape variety, ripeness, and experimental protocols (52).

**Figure 8:**
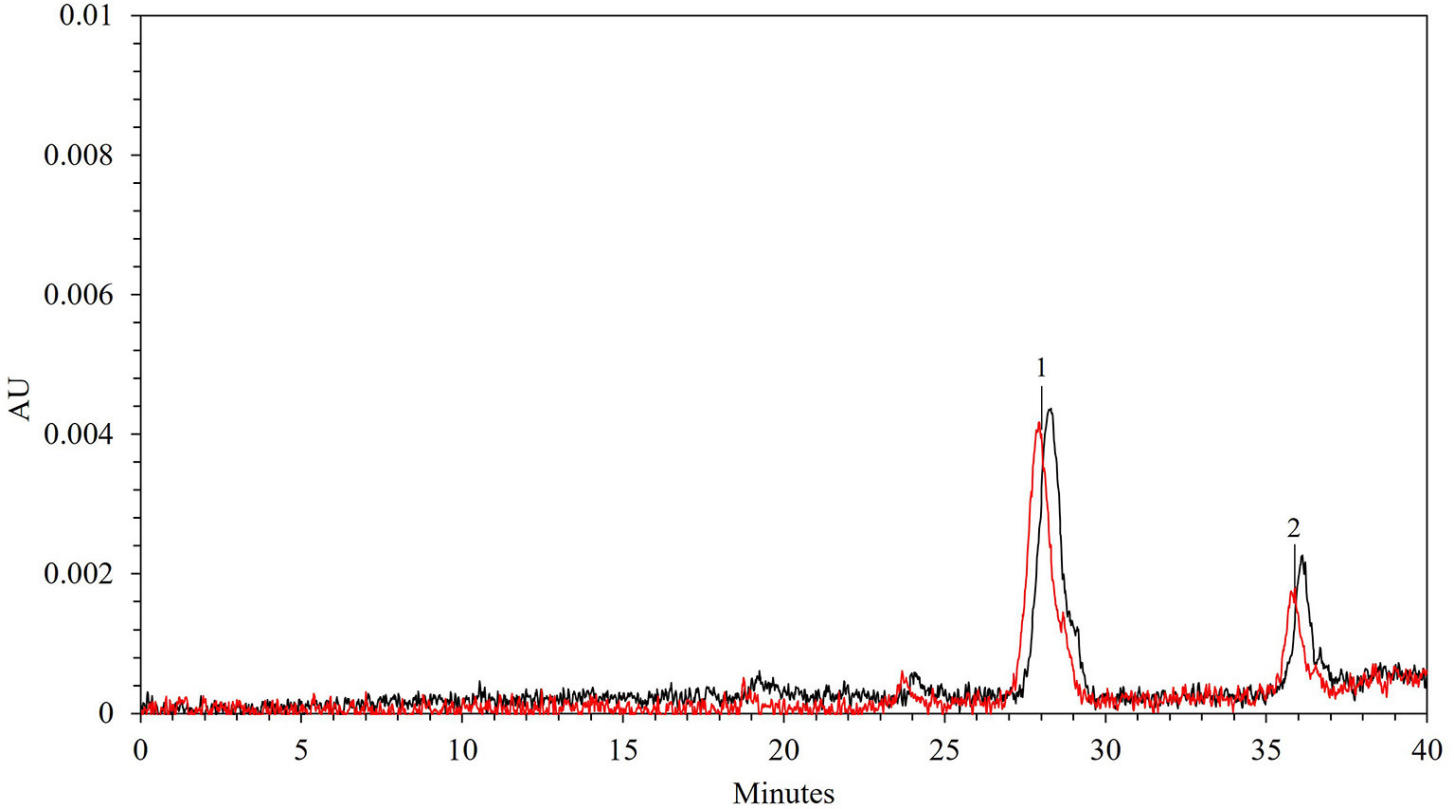
HPLC-PDA chromatograms of 50% (v/v) ethanol-water extracts obtained after 300 min of extraction at 50°C from untreated (red line) and PEF (E_opt_ = 4.6 kV/cm; W_T,opt_ = 20 kJ/kg)-treated (black line) red grape pomace. Peak identification: (1) peonidin 3-O-glucoside; (2) unidentified compound.

In Figure 8, the HPLC anthocyanin profiles of the extracts obtained from untreated and PEF-treated grape pomace under optimal conditions, are compared. As it can be seen, only one major peak corresponding to peonidin-3-O-glucoside (peak 1) with a concentration equal to 0.13 ± 0.004 mg/g_DW_ was clearly detected in the control extract at an elution time of 27.91 min, whereas one minor and unidentified compound (peak 2), was also detected at a higher elution time. Additionally, according to the results presented in Figure 7, the HPLC chromatogram also highlight demonstrated that PEF pre-treatment did not affect the type and number of the detected anthocyanins. This is consistent with results observed by other scientists on different red fruit by-products tissues, including those of red grape pomace, who found that the extracts obtained from untreated and PEF-treated grape pomace (19, 21, 22), press cakes of either blueberries (50) or sweet cherries (57) presented similar anthocyanin profiles, corroborating that no degradation occurred upon the application of PEF pre-treatments. However, it is worth noting that, the permeabilization of the cell membranes of grape pomace tissues upon PEF treatment, significantly enhanced the extractability of anthocyanin compounds, leading to a final concentration of peonidin-3-O-glucoside in the extract of 0.16 ± 0.002 mg/g_DW_, which was 23% higher than that detected in the control extract. A similar increment ranged between 5 and 20% in the extraction yield of peonidin 3-O-glucoside was also observed by Brianceau et al. (21) upon PEF-assisted extraction of grape pomace.

## 4. Conclusions

The results obtained in this study have demonstrated that the optimization of the processing conditions involved in the PEF-assisted extraction process, allowed achieving a high level of cell membrane permeabilization (*Z*_*p*_) of grape pomace tissue, thus intensifying the extractability of high-value-added compounds, such as total phenolic content (TPC, +15%), flavonoid content (FC, +60%), total anthocyanin content (TAC, +23%), tannin content (TC, +42), and, consequently, improving the antioxidant power (FRAP, +31) of the extracts.

The effects of independent factors, such as electric field strength and energy input for PEF, and ethanol concentration, extraction temperature and diffusion time for SLE, on the corresponding response variables (*Z*_*p*_ for PEF, and TPC, FC, TAC, TC and FRAP for SLE), were evaluated using FC-CCD and response surface methodology. The variables were significant and the adopted the second-order polynomial model and 2FI model accurately predicted the experimental values of the response variables for PEF pre-treatment and SLE step, respectively.

The HPLC analyses confirmed that PEF pre-treatment significantly improved the extraction yield of phenolic compounds and anthocyanins, namely epicatechin, p-coumaric acid, chlorogenic acid, gallic acid, and peonidin 3-O-glucoside, with no evidence of degradation of individual compounds due to PEF application.

The obtained results confirm the potential of PEF technology to promote the valorization of grape processing by-products allowing to intensify the extractability of a greater diversity of valuable compounds. Therefore, they encourage further investigations of the PEF-assisted extraction process at a larger scale in order to validate the results achieved in the present study as well as to evaluate the economic and environmental benefits derived from the implementation of this novel extraction technique against the conventional SLE process.

## Data availability statement

The raw data supporting the conclusions of this article will be made available by the authors, without undue reservation.

## Author contributions

GP and GF contributed conception, design of the study, and supervised the study. SC was in charge of performing chemical, statistical analysis, and wrote the first draft of the manuscript. GP and SC performed the experiments. All authors contributed to manuscript revision, read, and approved the submitted version.

## References

[1] Grassi F de Lorenzis G. Back to the origins: background and perspectives of grapevine domestication. Int J Mol Sci. (2021) 22:518. 10.3390/ijms22094518 33926017 PMC8123694

[2] Carpentieri S Ferrari G Pataro G. Optimization of pulsed electric fields-assisted extraction of phenolic compounds from white grape pomace using response surface methodology. Front Sustain Food Syst. (2022) 6:854968. 10.3389/fsufs.2022.854968 PMC10063923 37006934

[3] Beres C Costa GNS Cabezudo I da Silva-James NK Teles ASC Cruz APG. Towards integral utilization of grape pomace from winemaking process: A review. Waste Management. (2017) 68:581–94. 10.1016/j.wasman.2017.07.017 28734610

[4] Pataro G Carullo D Ferrari G. Innovative processes for the extraction of bioactive compounds from winery wastes and by-products. Imp Sust Viticult Winemaking Prac. (2022) 1:281–303. 10.1016/B978-0-323-85150-3.00004-9

[5] Gubitosa J Rizzi V Laurenzana A Scavone F Frediani E Fibbi G. The “end life” of the grape pomace waste become the new beginning: the development of a virtuous cycle for the green synthesis of gold nanoparticles and removal of emerging contaminants from water. Antioxidants. (2022) 11:994. 10.3390/antiox11050994 35624858 PMC9137750

[6] Kokkinomagoulos E Kandylis P. Sustainable Exploitation of By-Products of Vitivinicultural Origin in Winemaking. Basel: MDPI AG (2020).

[7] Fortified Wine Market Size,. Share Trends Analysis Report By Product (Port Wine, Vermouth, Sherry), By Distribution Channel (Pub, Bars & Restaurants, Internet Retailing, Liquor Stores, Supermarkets), By Region, Segment Forecasts, 2022 – 2028. (2023). Available online at: https://www.giiresearch.com/report/grvi1092424-fortified-wine-market-size-share-trends-analysis.html (accessed January 20, 2023).

[8] Erinle TJ Oladokun S MacIsaac J Rathgeber B Adewole D. Dietary grape pomace – effects on growth performance, intestinal health, blood parameters, and breast muscle myopathies of broiler chickens. Poult Sci. (2022) 101:101519. 10.1016/j.psj.2021.101519 34794081 PMC8605297

[9] Antonić BA Jančíková S Dordević D Tremlová B. Grape pomace valorization: a systematic review and meta-analysis. Foods. (2020) 9:1627. 10.3390/foods9111627 33171832 PMC7695143

[10] Coelho MC Pereira RN Rodrigues AS Teixeira JA Pintado ME. The use of emergent technologies to extract added value compounds from grape by-products. Trends Food Sci Technol. (2020) 106:182–97. 10.1016/j.tifs.2020.09.02833740601

[11] Merkyte V Longo E Windisch G Boselli E. Phenolic compounds as markers of wine quality and authenticity. Foods. (2020) 9:785. 10.3390/foods9121785 33271877 PMC7760515

[12] Martins IM Macedo GA Macedo JA. Biotransformed grape pomace as a potential source of anti-inflammatory polyphenolics: effects in Caco-2 cells. Food Biosci. (2020) 35:100607. 10.1016/j.fbio.2020.10060735334833

[13] Cisneros-Yupanqui M Lante A Mihaylova D Krastanov AI Rizzi C. The α-amylase and α-glucosidase inhibition capacity of grape pomace: a review. Food Bioproc Tech. (2022) 30:1–3. 10.1007/s11947-022-02895-0 36062030 PMC9427156

[14] Meini MR Cabezudo I Galetto CS Romanini D. Production of grape pomace extracts with enhanced antioxidant and prebiotic activities through solid-state fermentation by *Aspergillus niger* and *Aspergillus oryzae*. *Food Biosci*. (2021) 42:101168. 10.1016/j.fbio.2021.101168

[15] Rasul MG. Conventional extraction methods use in medicinal plants, their advantages and disadvantages. Int. J. Basic Sci. Appl. Comput. (2018) 2:1–14.

[16] Zhang QW Lin LG Ye WC. Techniques for extraction and isolation of natural products: a comprehensive review. Chin Med. (2018) 13:1–26. 10.1186/s13020-018-0177-x 29692864 PMC5905184

[17] Carpentieri S Soltanipour F Ferrari G Pataro G Dons F. Emerging green techniques for the extraction of antioxidants from agri-food by-products as promising ingredients for the food industry. Antioxidants. (2021) 10:1417. 10.3390/antiox10091417 34573049 PMC8471374

[18] Carullo D Pataro G Donsì F Ferrari G. Pulsed electric fields-assisted extraction of valuable compounds from arthrospira platensis: effect of pulse polarity and mild heating. Front Bioeng Biotechnol. (2020) 8:551272. 10.3389/fbioe.2020.551272 33015015 PMC7498763

[19] Barba FJ Brianceau S Turk M Boussetta N Vorobiev E. Effect of alternative physical treatments (ultrasounds, pulsed electric fields, and high-voltage electrical discharges) on selective recovery of bio-compounds from fermented grape pomace. Food Bioproc Tech. (2015) 8:1139–48. 10.1007/s11947-015-1482-3

[20] Medina-Meza IG Barbosa-Cánovas G. V. Assisted extraction of bioactive compounds from plum and grape peels by ultrasonics and pulsed electric fields. J Food Eng. (2015) 166:268–75. 10.1016/j.jfoodeng.2015.06.012

[21] Brianceau S Turk M Vitrac X Vorobiev E. Combined densification and pulsed electric field treatment for selective polyphenols recovery from fermented grape pomace. Innov Food Sci Emerg Technol. (2015) 29:2–8. 10.1016/j.ifset.2014.07.010

[22] Corrales M Toepfl S Butz P Knorr D Tauscher B. Extraction of anthocyanins from grape by-products assisted by ultrasonics, high hydrostatic pressure or pulsed electric fields: a comparison. Innov Food Sci Emerg Technol. (2008) 9:85–91. 10.1016/j.ifset.2007.06.002

[23] Cholet C Delsart C Petrel M Gontier E Grimi N. L'Hyvernay A, Ghidossi R, Vorobiev E, Mietton-Peuchot M, Gény L. Structural and biochemical changes induced by pulsed electric field treatments on cabernet sauvignon grape berry skins: Impact on cell wall total tannins and polysaccharides. J Agric Food Chem. (2014) 62:2925–34. 10.1021/jf404804d 24617601

[24] Casazza AA Aliakbarian B de Faveri D Fiori L Perego P. Antioxidants from winemaking wastes: A study on extraction parameters using response surface methodology. J Food Biochem. (2012) 36:28–37. 10.1111/j.1745-4514.2010.00511.x

[25] Rajha HN El Darra N Vorobiev E Louka N Maroun RG. An environment friendly, low-cost extraction process of phenolic compounds from grape byproducts. optimization by multi-response surface methodology. Food Nutr Sci. (2013) 4:650–9. 10.4236/fns.2013.46084

[26] Melo PS Massarioli AP Denny C dos Santos LF Franchin M Pereira GE. Winery by-products: Extraction optimization, phenolic composition and cytotoxic evaluation to act as a new source of scavenging of reactive oxygen species. Food Chem. (2015) 181:160–9. 10.1016/j.foodchem.2015.02.087 25794735

[27] Caldas TW Mazza KEL Teles ASC Mattos GN Brígida AIS Conte-Junior CA. Phenolic compounds recovery from grape skin using conventional and non-conventional extraction methods. Ind Crops Prod. (2018) 111:86–91. 10.1016/j.indcrop.2017.10.012

[28] Thomareis AS Dimitreli G. Techniques Used for processed cheese characterization. Processed Cheese Science and Technology: Ingredients, Manufacture, Functionality, Quality, and Regulations. London: Woodhead Publishing. (2022).

[29] Kwiatkowski M Kravchuk O Skouroumounis GK Taylor DK. Response surface parallel optimization of extraction of total phenolics from separate white and red grape skin mixtures with microwave-assisted and conventional thermal methods. J Clean Prod. (2020) 251:119563. 10.1016/j.jclepro.2019.119563

[30] Medouni-Adrar S Boulekbache-Makhlouf L Cadot Y Medouni-Haroune L Dahmoune F Makhoukhe A. Optimization of the recovery of phenolic compounds from Algerian grape by-products. Ind Crops Prod. (2015) 77:123–32. 10.1016/j.indcrop.2015.08.039

[31] Donsì F Ferrari G Fruilo M Pataro G. Pulsed electric field-assisted vinification of aglianico and piedirosso grapes. J Agric Food Chem. (2010) 58:11606–15. 10.1021/jf102065v 21038868

[32] Frontuto D Carullo D Harrison SM Brunton NP Ferrari G Lyng JG. Optimization of Pulsed Electric Fields-Assisted Extraction of Polyphenols from Potato Peels Using Response Surface Methodology. Food Bioproc Tech. (2019) 12:1708–20. 10.1007/s11947-019-02320-z

[33] Carpentieri S Mazza L Nutrizio M Jambrak AR Ferrari G Pataro G. Pulsed electric fields- and ultrasound-assisted green extraction of valuable compounds from Origanum vulgare L. and Thymus serpyllum L Int J Food Sci Technol. (2021) 48:1–9. 10.1111/IJFS.15159/v2/response1

[34] Lee J Durst RW Wrolstad RE. Determination of total monomeric anthocyanin pigment content of fruit juices, beverages, natural colorants, and wines by the pH differential method: collaborative study. J AOAC Int. (2005) 88:1269–78. 10.1093/jaoac/88.5.1269 16385975

[35] Tempel AS. Tannin-measuring techniques - A review. J Chem Ecol. (1982) 8:1289–98. 10.1007/BF00987762 24414735

[36] Benzie IFF Strain JJ. The Ferric Reducing Ability of Plasma (FRAP) as a Measure of “Antioxidant Power”: The FRAP Assay. Anal Biochem. (1996) 239:70–6. 10.1006/abio.1996.0292 8660627

[37] Lee J Rennaker C Wrolstad RE. Correlation of two anthocyanin quantification methods: HPLC and spectrophotometric methods. Food Chem. (2008) 110:782–6. 10.1016/j.foodchem.2008.03.010

[38] Boussetta N Lebovka N Vorobiev E Adenier H Bedel-Cloutour C Lanoisellé JL. Electrically assisted extraction of soluble matter from chardonnay grape skins for polyphenol recovery. J Agric Food Chem. (2009) 57:1491–7. 10.1021/jf802579x 19173604

[39] Duba KS Casazza AA Mohamed H. ben, Perego P, Fiori L. Extraction of polyphenols from grape skins and defatted grape seeds using subcritical water: experiments and modeling. Food Bioproducts Proc. (2015) 94:29–38. 10.1016/j.fbp.2015.01.001

[40] Kostić DA Dimitrijević DS Mitić SS Mitić MN Stojanović GS Živanović A. Phenolic content and antioxidant activities of fruit extracts of *Morus nigra* L (Moraceae) from southeast serbia tropical. J Pharm Res. (2013) 12:105–10. 10.4314/tjpr.v12i1.17

[41] Dhull SB Kaur P Purewal SS. Phytochemical analysis, phenolic compounds, condensed tannin content and antioxidant potential in Marwa (*Origanum majorana*) seed extracts. Res Eff Technol. (2016) 2:168–74. 10.1016/j.reffit.2016.09.003

[42] Spigno G Tramelli L de Faveri DM. Effects of extraction time, temperature and solvent on concentration and antioxidant activity of grape marc phenolics. J Food Eng. (2007) 81:200–8. 10.1016/j.jfoodeng.2006.10.02131079623

[43] Niu Y Xiang Y. An Overview of Biomembrane Functions in Plant Responses to High-Temperature Stress. Front Plant Sci. (2018) 9:915. 10.3389/fpls.2018.00915 30018629 PMC6037897

[44] Medina-Plaza C Beaver JW Lerno L Dokoozlian N Ponangi R Blair T. Impact of temperature, ethanol and cell wall material composition on cell wall-anthocyanin interactions. Molecules. (2019) 24:350. 10.3390/molecules24183350 31540067 PMC6767090

[45] Gurtovenko AA Anwar J. Interaction of ethanol with biological membranes: the formation of non-bilayer structures within the membrane interior and their significance. J Phys Chem B. (2009) 113:1983–92. 10.1021/jp808041z 19199697

[46] Chew KK Ng SY Thoo YY Khoo MZ Wan Aida WM and Ho CW. Effect of ethanol concentration, extraction time and extraction temperature on the recovery of phenolic compounds and antioxidant capacity of Centella asiatica extracts. Int Food Res J. (2011) 18:571–8.

[47] Watrelot AA. Norton EL. Chemistry and reactivity of tannins in vitis spp: A review. Molecules. (2020) 25:110. 10.3390/molecules25092110 32365968 PMC7248762

[48] Monrad JK Howard LR King JW Srinivas K Mauromoustakos A. Subcritical solvent extraction of procyanidins from dried red grape pomace. J Agric Food Chem. (2010) 58:4014–21. 10.1021/jf9028283 20020688

[49] Pereira DTV Tarone AG Cazarin CBB Barbero GF Martínez J. Pressurized liquid extraction of bioactive compounds from grape marc. J Food Eng. (2019) 240:105–13. 10.1016/j.jfoodeng.2018.07.019

[50] Pataro G Bobinaite R Bobinas C Šatkauskas S Raudonis R Visockis M. Improving the extraction of juice and anthocyanins from blueberry fruits and their by-products by application of pulsed electric fields. Food Bioproc Tech. (2017) 10:1595–605. 10.1007/s11947-017-1928-x26345006

[51] de Sales NFF da Costa LS Carneiro TIA Minuzzo DA Oliveira FL Cabral LMC. El-Bacha T. Anthocyanin-rich Grape Pomace Extract (*Vitis vinifera* L) from wine industry affects mitochondrial bioenergetics and glucose metabolism in human hepatocarcinoma HepG2 cells. Molecules. (2018) 23:611. 10.3390/molecules23030611 29518033 PMC6017946

[52] Castellanos-Gallo L Ballinas-Casarrubias L Espinoza-Hicks JC Hernández-Ochoa LR Muñoz-Castellanos LN Zermeño-Ortega MR. Grape pomace valorization by extraction of phenolic polymeric pigments: a review. Processes. (2022) 10:469. 10.3390/pr1003046929132780

[53] Atak A Göksel Z Yilmaz Y. Changes in Major Phenolic Compounds of Seeds, Skins, and Pulps from Various Vitis spp. and the effect of powdery and downy mildew diseases on their levels in grape leaves. Leaves. (2021) 10:2554. 10.3390/plants10122554 34961024 PMC8703439

[54] Calderón-Oliver M Ponce-Alquicira E. Fruits: a source of polyphenols and health benefits. Nat Artif Flav Agents Food Dyes. (2018)189–228. 10.1016/B978-0-12-811518-3.00007-7

[55] Bobinaite R Pataro G Lamanauskas N Šatkauskas S Viškelis P Ferrari G. Application of pulsed electric field in the production of juice and extraction of bioactive compounds from blueberry fruits and their by-products. J Food Sci Technol. (2015) 52:5898–905. 10.1007/s13197-014-1668-0 26345006 PMC4554608

[56] Antoniolli A Fontana AR Piccoli P Bottini R. Characterization of polyphenols and evaluation of antioxidant capacity in grape pomace of the cv. Malbec Food Chem. (2015) 178:172–8. 10.1016/j.foodchem.2015.01.082 25704698

[57] Pataro G Carullo D Bobinaite R Donsì G Ferrari G. Improving the extraction yield of juice and bioactive compounds from sweet cherries and their by-products by pulsed electric fields. Chem Eng Trans. (2017) 57:1717–22. 10.3303/CET1757287

