# Supplementary material for: Pulsed electric fields-assisted extraction of valuable compounds from red grape pomace: Process optimization using response surface methodology
Source: Front Nutr. 2023 Mar 17;10:1158019. doi: 10.3389/fnut.2023.1158019 (PMC10063923; doi:10.3389/fnut.2023.1158019)
Supplement: Supplementary file 1 [file Data_Sheet_1.docx]

Supplementary Material

Pulsed electric fields-assisted extraction of valuable compounds from red grape pomace: process optimization using response surface methodology

**Serena Carpentieri^1^, Giovanna Ferrari^1,2^, Gianpiero Pataro^1*^**

^1^Department of Industrial Engineering, University of Salerno, Fisciano, Salerno, Italy

^2^ProdAl Scarl c/o University of Salerno, Fisciano, Salerno, Italy.

*** Correspondence:**Corresponding Author: Gianpiero Pataro

E-mail address: [gpataro@unisa.it](mailto:gpataro@unisa.it)

# Effect of PEF pre-treatment on electrical impedance of grape pomace tissue

Figure S1 depicts the average trend of the absolute value of the complex impedance for untreated (control) and PEF-treated red grape pomace varying the frequency applied and at the combinations, obtained from the RSM study and previously reported in Table 1, of field strength E (0.5-5 kV/cm) and energy input W_T_ (1-20 kJ/kg).

Results obtained from the measurements of complex impedance of red grape pomace tissue before and after PEF treatment, carried out to quantify the cell permeabilization degree (1), show that the impedance of intact plant tissue is frequency dependent. In particular, at low frequency range the cell membrane acts as a dielectric having a weak complex conductivity, as increasing the frequency the membrane gradually becomes more permeable to the electric field, while at very high frequency range, the membrane is completely shorted out, showing an electrical transparency (2,3).

The application of a PEF treatment significantly induced a decrement in the impedance value due to the loss of the capacitive properties of the cell membrane upon the permeabilization induced by the PEF application.

In particular, for the same energy input, as the field strength applied increased, the cell membrane damage increased, which was more pronounced for field strength above 0.5 kV/cm. On the other hand, for any field strength applied, the higher the energy input the lower the absolute value of the complex impedance, especially for values greater than 10.5 kJ/kg. These results highlight the capability of PEF to induce an increment in the cell membrane permeability depending on the treatment intensity applied, nevertheless the field strength is demonstrated to be the parameter that mostly affected the cell permeability. Based on the obtained results, the cell disintegration index (*Zp*) was determined as described in the section 2.3 of the manuscript, and used as a reliable indicator to determine the extent of cell membrane damage of grape pomace tissue induced by PEF treatment, as well as the optimal PEF treatment conditions to be applied for further experiments.


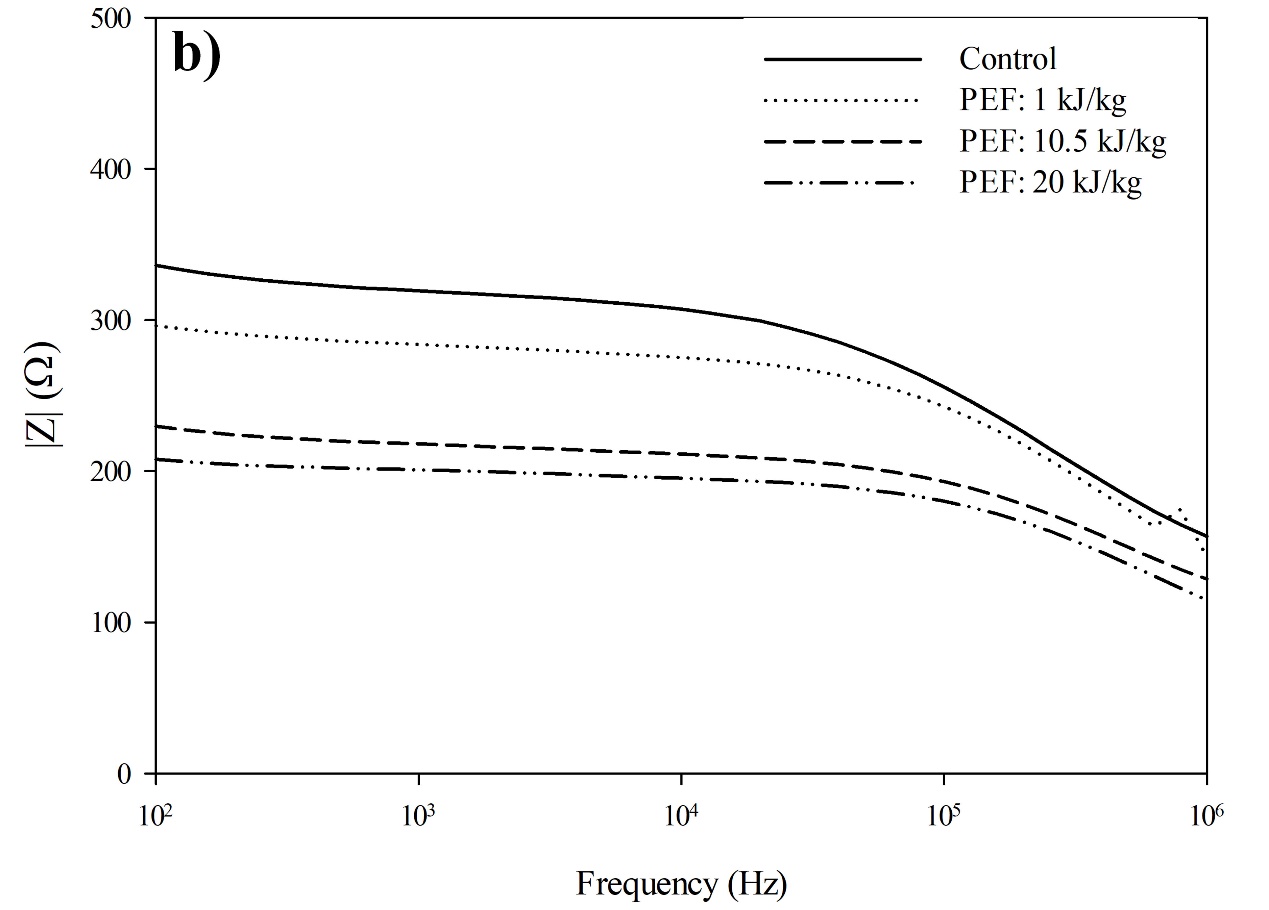

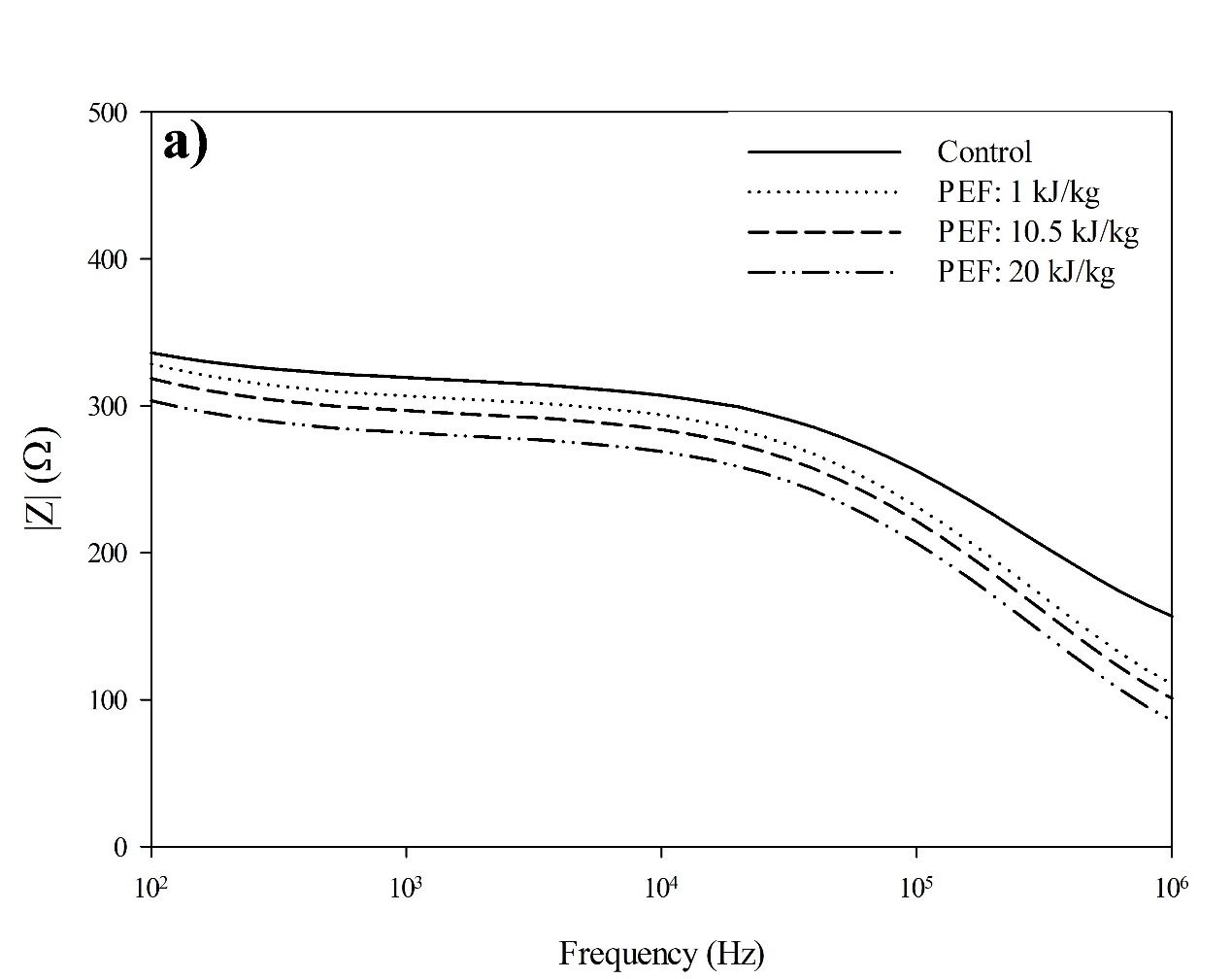


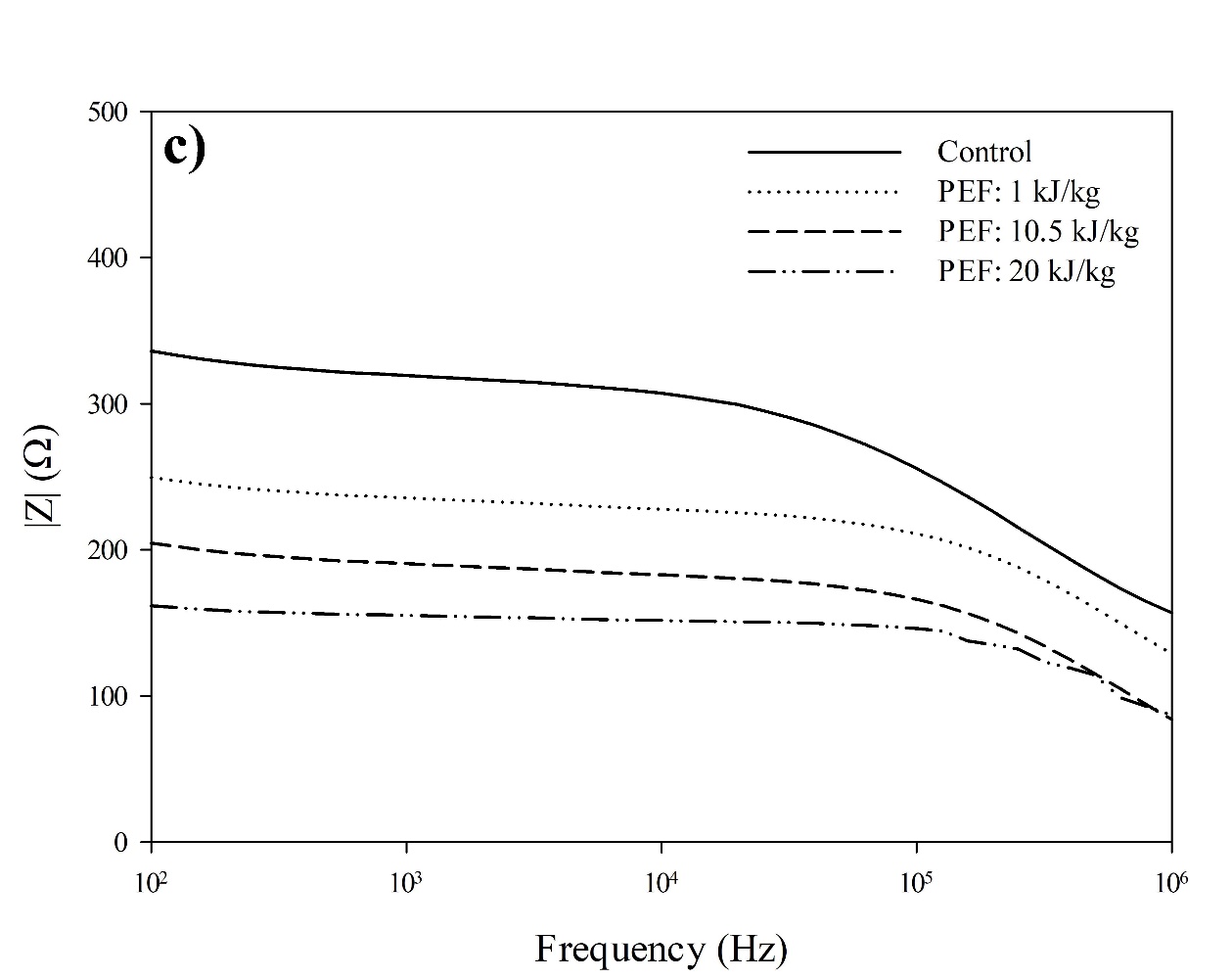


Figure S1. Frequency dependency of the absolute value of complex impedance (|Z|) for red grape pomace before (Control) and after PEF treatment of different energy inputs. Field strength set at (a)

0.5 kV/cm, (b) 2.75 kV/cm, and (c) 5 kV/cm.

**References**

1. Donsì F, Ferrari G, Pataro G. Applications of Pulsed Electric Field Treatments for the Enhancement of Mass Transfer from Vegetable Tissue. *Food Engineering Reviews* (2010) 2:109–130. doi: 10.1007/s12393-010-9015-3

2. Khoshmanesh K, Nahavandi S, Baratchi S, Mitchell A, Kalantar-zadeh K. Dielectrophoretic platforms for bio-microfluidic systems. *Biosens Bioelectron* (2011) 26:1800–1814. doi: 10.1016/j.bios.2010.09.022

3. Battipaglia G, De F, Prodal V, Donsì F, Prodal GF, Pataro G. ENHANCEMENT OF POLYPHENOLS EXTRACTION FROM INVOLUCRAL BRACTS OF ARTICHOKES.
